# Development of a paediatric model of diffuse traumatic brain injury in ferrets

**DOI:** 10.1038/s41598-026-37303-6

**Published:** 2026-01-23

**Authors:** Justin L. Krieg, Carl Hooper, Hasini Kapuwelle, Rebecca P. George, Rebecca J. Hood, William T. O’Brien, Stuart J. McDonald, Anna V. Leonard, Renée J. Turner, Frances Corrigan

**Affiliations:** 1 Translational Neuropathology Laboratory, School of Pharmacy and Biomedical Sciences, Adelaide University, Adelaide, Australia; 2 Department of Neuroscience, School of Translational Medicine, Monash University, 99 Commercial Road, Melbourne, VIC Australia

**Keywords:** Axonal injury, Diffuse traumatic brain injury, Ferret, Motor, Cognition, Paediatric, Medical research, Neurology, Neuroscience

## Abstract

Traumatic brain injury (TBI) is a leading cause of death and disability in children, especially those under five, with younger children more vulnerable to persistent cognitive and neuropsychological effects due to disrupted brain development. Paediatric brains are biomechanically more susceptible to diffuse axonal injury due to anatomical differences, with axonal injury observed in up to 80% of hospitalized children. Despite this, many preclinical TBI models focus on focal injuries, whereas to better model the evolution of axonal injury in the paediatric brain, clinically-relevant diffuse pre-clinical models are required. In this study gyrencephalic paediatric ferrets (2–3 months) equivalent to a 3–5-year-old child were injured with the Closed Head Impact Model of Engineered Rotational Acceleration (CHIMERA), and the axonal injury response and resultant motor and cognitive deficits examined to 72 h post-injury. Injury was associated with extensive axonal injury, as detected via both the amyloid precursor protein (APP) for transport disruption and neurofilament light (NFL) for neurofilament integrity, in key structures including the corpus callosum, fornix and cortical white matter. Serum GFAP was increased within 30 min of injury, returning to baseline by 72 h, with NFL elevated from 24 h to 72 h post-injury. Injured ferrets had deficits in balance, working memory and spatial memory. The adaptation of the CHIMERA model to paediatric ferrets provides the opportunity to investigate factors driving axonal pathology post-injury and how these interact with neurodevelopment.

## Introduction

 Traumatic brain injury (TBI) is considered to be the leading cause of death and disability in children^1, 2^. In the US alone, ∼500,000 children and adolescents under the age of 14 sustain a TBI that results in a visit to an emergency department at a cost exceeding $1 USD billion annually^2, 3^. Incidence rates are not uniform throughout the paediatric population with the highest rate in 0–5-year-olds^4, 5, 6^. Within this population mechanism of injury differs, with non-accidental head injury higher in infants, whilst head injury in early childhood is associated with falls and motor vehicle accidents. Notably, younger children (3–7 yrs old) also show higher rates of persistent cognitive and neuropsychological outcomes than older children (8–11 yrs old)^7, 8^, given that injury may alter the trajectory of normal brain development^9^. Considering this, the need for further paediatric TBI research, particularly in the early childhood age range is required.

It is well established that adults and children respond differently to a mechanical insult, given their anatomical differences. This includes a higher head to body ratio, and less developed neck muscles increasing the biomechanical load placed onto the paediatric brain^10^. Furthermore, skull thickness is generally thinner and less capable of withstanding force^11^. Clinically, this is reflected by the increased incidence of skull fracture within the paediatric population^10^. Despite this, focal injuries and contusions are less prevalent in paediatric populations, with computed tomography (CT)-negative patients indicative of axonal injury, almost double that of adults^12^. Indeed, the greater plasticity of the skull may also lead to greater shear forces to the underlying brain^13^, which may be further exacerbated by the increased water content with the paediatric brain, due to smaller amount of myelin. Diffuse axonal injury is thought to be present in as many as 80% of children hospitalised for TBI^14^, contrasting the 48% shown for adults^15^. Given that it is well established in adults that axonal injury even in mild TBI is linked to subacute and chronic neurological deficits^16, 17^, this axonal injury burden could have long-lasting consequences.

Despite the prevalence of diffuse axonal injury, pre-clinical paediatric TBI models predominantly use the controlled cortical impact model (CCI) model^18^ to produce a focal contusion. In CCI much of the axonal injury is associated with neuronal death, rather than shearing forces^19, 20^. Hence to model pure diffuse injury several different approaches have been taken to adapt adult injury models to the paediatric population. In rodents these include the Marmarou weight drop model, where a weight is dropped onto a metal disc affixed to the skull^21, 22, 23, 24^, the closed skull CCI injury with the impactor either directed to the midline^25, 26^ or to one side^27, 28^, or the midline fluid percussion injury (mFPI) model^29, 30, 31^. However, there are inherent limitations to each of these models, including limited head movement in the closed head CCI models, unlike clinical TBI where head movement is unrestricted^32^. The weight drop models have also been associated with high mortality rates of 48–58% which does not fit the clinical paediatric injury picture^21, 33^, whilst mFPI requires a craniotomy, which can by itself cause damage to the underlying brain^34^, and does not represent the predominance of closed head injury seen clinically.

In addition, the rodent brain is lissencephalic with limited white matter^35^, which may alter the way the brain responds to mechanical impact^36^. To fill this gap, white matter-rich, gyrencephalic piglets have been used to model paediatric TBI, with an adaption of the HYGE model^37, 38, 39, 40^. Many of these studies were conducted in 3–5 day old piglets, modelling TBI in young infants (< 3 months)^38, 41, 42, 43^, with relatively few examining the effects in older toddler equivalent (4–6 week) pigs^37, 40, 44, 45^. Indeed, the axonal injury response and effects on behaviour in this age group have only been examined to 6 h post-injury^46^, so little is known about the evolution of axonal injury and the resultant motor and cognitive symptoms beyond this. As studies utilising pigs are limited by their expense and the need for specialised husbandry and surgical expertise^47^, ferrets offer an alternative, given their gyrencephalic brain and small size. Early neurodevelopment in ferrets is equivalent to a preterm human infant reaching a maturation of newborn infant by ~ 3–4 weeks of age^48^, meaning that slightly older animals can be used to mimic the response in children under 5. Indeed, the 2–3-month-old ferret is suggested to be equivalent in terms of neurodevelopment to human age of 3–5^49, 50^, in line with 4–6 week old pigs^37, 40, 44, 45^ and postnatal day (PD)17–30 mice or rats^51^.

The closed head impact model of engineered acceleration rotation (CHIMERA) was recently adapted for adult ferrets showing graded axonal injury^52^. Here we utilised 2–3-month-old ferrets to mimic injury in early childhood, with examination of the evolution of axonal injury and microglial response to 72 h post-injury and resultant effects on motor and cognitive function.

## Methods

### Study design

Studies were designed and reported in accordance with the *ARRIVE (Animal Research: Reporting of In Vivo Experiments)* guidelines. All studies were also in accordance with the National Health and Medical Research Council of Australia’s Code for the Care and Use of Animals for Scientific Purposes and procedures were approved by the South Australian Health and Medical Research Institute Animal Ethics Committee (SAHMRI AEC; approval no: SAM 21–074). Ferrets (Mustela furo) were purchased from Animalatic (Roxburgh Park, Melbourne) and arrived on-site 2-weeks before any procedures to acclimatise and were all included within the study.

Three studies were conducted: (1) 24 h survival time-point for immunohistochemical analyses; (2) 72 h survival time-point for immunohistochemical analyses; (3) small pilot study investigating intracranial pressure (ICP). In the 24 h study *n* = 13 paediatric male ferrets (Average age: PND77 [65–94]; Average weight: 0.66 kg [0.49–0.89 kg]), were randomly allocated via random number generator to sham (*n* = 5) and injury (*n* = 8) groups. The 72 h and pilot ICP studies were then conducted 1 year later with mixed sex ferrets with an average age of PND: 91[71–105] and average weight 0.67 kg [0.50–0.94 kg]. Ferrets were randomly allocated to sham or injury groups with *n* = 23 in the 72 h study (Sham *n* = 10; 6 F:4 M; TBI *n* = 13; 6 F:7 M) and *n* = 8 in the pilot ICP study (Sham *n* = 4 2 F:2 M; TBI *n* = 4 2 F:2 M). Male ferrets were slighter larger than female ferrets in this cohort (0.78 kg [0.65–0.92 kg] vs. (0.62 kg [0.50–0.74 kg]). Animals were group housed by sex in cages of 2–4 ferrets.

### Anaesthetisation and preparation

Animals were placed into a large induction chamber pre-filled with 5% isoflurane (1 L/min 100% oxygen) for approximately 4 min, or until a loss of palpebral and pedal reflexes. The first *n* = 6 animals in the 24 h cohort (*n* = 2 sham; *n* = 4 injury) were intubated with a non-cuffed 2.0 endotracheal tube, but later animals (24 h: *n* = 3 sham; *n* = 10 injury; 72 h: all) were maintained using a large feline mask. The small size of paediatric animals made intubation impractical and added extra time under anaesthetic, with little benefits. In both instances, they were maintained between 2 and 3% isoflurane (1 L/min 100% oxygen). Animals had continuous monitoring of oxygen saturation, and heart rate (HR) via a pulse oximeter applied to the shaved tail of the animal (Phillips MP30). A continuous feedback rodent warmer was used to maintain animals at 38 °C (rodent warmer x1; Stoelting, USA). Animals were given subcutaneous saline (0.9%; 10 ml across two sites) to maintain hydration, and buprenorphine (0.015 mg/kg; Troy Laboratories, Australia) to provide post-operative analgesia 10 min before injury.

### TBI induction

Diffuse TBI was induced using the CHIMERA model, where a pneumatically driven piston strikes the dorsal skull, as previously described^52^. Ferrets were placed on the injury device in a supine position, secured in place with straps around the abdomen and shoulder, and ears aligned with the crosshairs on the device to ensure consistent injury location. Impact was set at 22 J for one 24 h TBI ferret and subsequently decreased to 17 J for all other injured ferrets. Sham animals were placed on the injury device, aligned, and secured with Velcro straps, but the device was not fired. Animals were maintained under anaesthesia for 30 min, with a serum sample collected and then recovered with 1 L/min of 100% oxygen. Animals were then returned to an individual cage with *ad libitum* access to food and water, with monitoring every 30 min until returned to normal behaviour (approximately 2 h following anaesthesia cessation).

### Intracranial and mean arterial pressure measurement

Intracranial pressure was assessed at 24 h post-TBI. Animals were re-anaesthetised and placed in the prone position. A burr hole was drilled into the right parietal bone followed by insertion of a skull bolt. A Codman microsensor ICP probe (Codman Neuro, DePuy Synthes, Australia) was inserted through the bolt into the epidural space and ICP recorded for 30 min using LabChart Pro (ADInstruments v8). During ICP measurement, mean arterial pressure was simultaneously measured using a non-invasive tail cuff and arterial and venous blood sampled for blood gas analysis (CD8 + cartridges, i-Stat, Abbott, Australia).

### Functional assessment

 Ferrets in the 72 h cohort underwent a behavioural battery to assess motor and cognitive outcomes following injury (Table 1). Motor outcome was assessed via the ladder-walk and open field and cognition on the quadrant maze and puzzlebox, as previously described^53^. All behavioural tests were recorded via Webcam and then analysed by a blinded investigator to prevent bias.

**Table 1:**
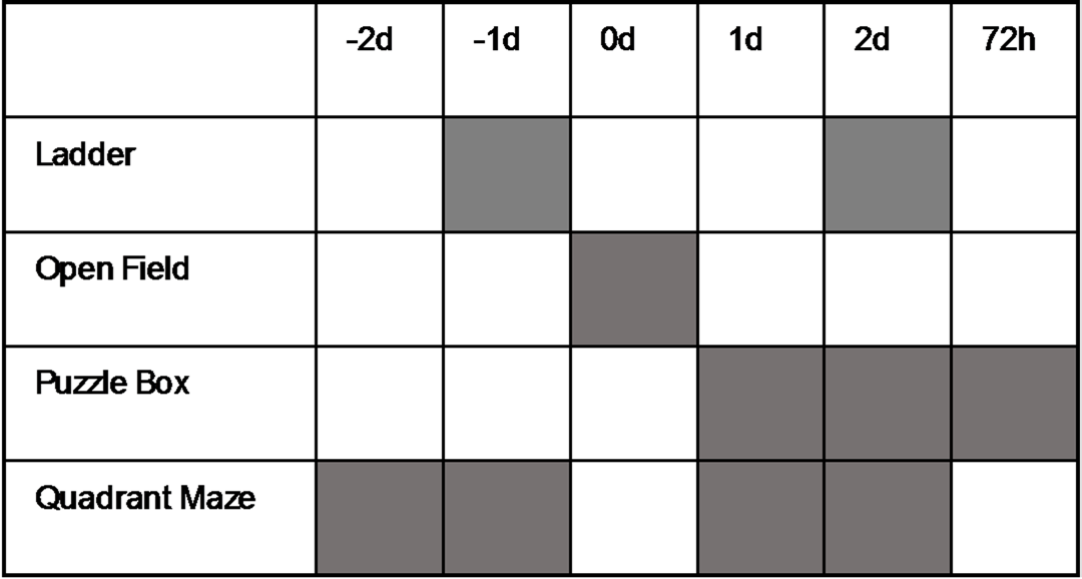
Timeline of the behavioural assay. Grey squares indicate when the assay was performed.

### Ladder walk

Ferrets were required to cross a 1.8 m ladder with 24 rungs spaced 7 cm apart, with ferrets given one familiarisation trial prior to injury, and then tested on day 2 post-injury. Each ferret was given one trial, with time to cross and faulty steps (limb slips through the ladder) assessed. Given the diffuse nature of the injury, faults across all four limbs were assessed and presented as percentage of faulty steps.

### Open field

To assess the general locomotor activity ferrets were placed in a 180 cm x 180 cm box for 3 min at 24 h post injury as previously reported in rodents^54^. The distance travelled was analysed using AnyMaze (v7.2; Stolting, USA).

### Quadrant maze

Spatial memory and cognitive flexibility were assessed on the Quadrant Maze. This is a 180 cm x 180 cm arena with curtained compartments in each corner, marked by a shape for visual differentiation. A milk reward was randomly located in one of the corners, with ferrets trained prior to injury over four trials to learn the location. Following injury ferrets were first tested on their ability to remember this location (T5), and then on their ability to learn a new location (T6 and T7). T6 was conducted 1 h after T5, and T7, 24 h later.

### Puzzle box

**Fig. 1:**
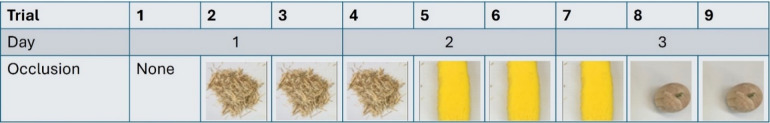
Outline of the sequence of trials and the obstruction used for the puzzlebox.

Problem solving, short- and long-term memory was assessed by the puzzlebox (100 cm x 30 cm x 60 cm), based on the task as previously used in mice^55^. The box has a start box connected to a goal box by a polyvinyl chloride (PVC) pipe that allows ferrets to move between the two compartments as depicted in Krieg et al. 2025 ^54^. This pipe is obstructed with increasingly difficult materials (straw, then a foam block and finally a wooden block) to test the ability of the ferrets to learn how to remove the obstruction and then how well they remember this strategy (Fig. 1). Ferrets are given three trials a day 1 h apart. On day 1, in the first trial the pipe is left open to ensure that ferrets can traverse between the two chambers. In trial 2 and 3 the pipe is obstructed with straw. On day 2, the pipe is again obstructed with straw for trial 4, and then with a foam block for trial 5 and 6. Finally on day 3, trial 7 has the same foam block obstruction and in trials 8 and 9 the pipe is again obstructed by a wooden block. Thus trials 2, 5 and 8 test problem solving as this is the ferret’s first exposure to a particular type of obstruction, trials 3, 6 and 9 test short-term memory as ferrets are exposed to the same obstruction 1 h later and trials 4 and 7 test long-term memory as ferrets face the same obstruction 24 h later. Performance is assessed via latency to the goal zone.

## Histology

### Perfusion

At the designated endpoint, animals were anaesthetised with isoflurane, as described above. A small incision was then made in the skin over the cephalic vein on the forearm with a scalpel and a 0.8 ml sample of blood was collected, separated for serum, and stored at −80 °C. The catheter was also used to administer heparin (1,000IU; Pfizer, USA), with ferrets then humanely killed via exsanguination and transcardial perfusion with 1 L of 10% formalin. Brains were removed and stored in 10% formalin for 2–4 weeks.

### Immunofluorescence

Brains were sectioned into 10 mm slices, embedded in paraffin wax and 5 μm sections were cut at.

**Fig. 2:**
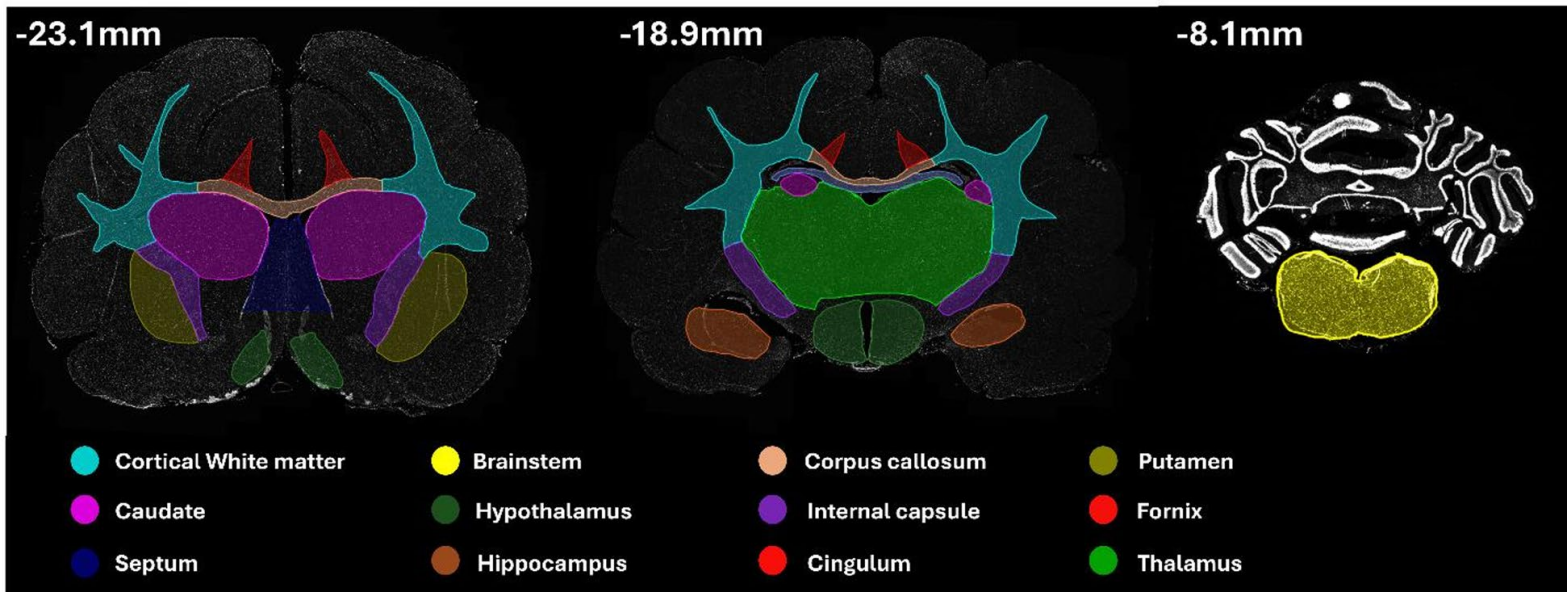
Regions of interest used for analysis, encompassing white and grey matter structures across three antero-posterior levels.

−17.1 mm, −18.9 mm and − 8.1 mm relative to the occipital crest to capture regions of interest. These included the white matter structures corpus callosum, cingulum, cortical white matter, fornix, internal capsule and brainstem, alongside grey matter structures in the caudate, putamen, hippocampus, hypothalamus and thalamus (Fig. 2). Cortical white matter, the corpus callosum, cingulum, hypothalamus, caudate, and internal capsule were analysed across two sections per brain, whereas the septum, brainstem, hippocampus, fornix, putamen, and thalamus were analysed across one section per brain. Multiplex immunofluorescence with APP, NFL, and IBA-1 (Table 2) was then used to examine axonal injury and the microglial response.

Slides were dewaxed with heat and placed in xylene for 2 × 3mins and then ethanol for 2 × 3mins. Heat-induced antigen retrieval was performed in 10 mM citrate buffer (pH 6.0). Slides immersed in the buffer were brought to boiling temperature in a microwave, then maintained in the microwave at a medium–low setting for a further 10mins. Following cooling, sections were blocked with normal goat serum (NGS; 1:30 in phosphate-buffered saline- PBS; Cat No: 16210064; Gibco, New Zealand) for 30 min, and primary antibodies applied overnight. The following day, fluorescent-tagged secondary antibodies were applied at a dilution ratio of 1:400 in a darkened humidity chamber. DAPI (4′,6-diamidino-2-phenylindole; 1:100,000; D9542; Sigma-Aldrich) was then applied to the tissue for 5 min, after which slides were cover slipped with FluromountG (Cat No: 00–4958-02; Thermo Fisher). Slides were imaged in a Zeiss AxioScan Z1 (Carl Zeiss AG, Germany) optimised to detect DAPI and the fluorophores at emission wavelength of 488nm, 555nm and 647nm at 20x magnification. Slides were then placed in PBS to allow the mounting media to dissolve and for coverslips to be removed. Following this, neat NewBlot nitro 5X stripping buffer (Cat No: LCR-928–40030; Li-Cor Biosciences; USA) was applied to the tissue for 15mins, as previously optimised^53, 56^. Slides were washed in PBS, and the second round of primary antibodies were applied, followed by the appropriate secondary, and coverslipping. A negative control (omitting the primary antibody) and single-antibody controls were also included to verify staining specificity. Slides were scanned, and the two images stitched together using VALIS (v1.0.4) such that all channels were visible in QuPath (v0.5.1)^57^, as previously described^53^. All files imported into QuPath used the `Mask image name’ feature to ensure the assessor was blinded for analysis.

**Table 2:** Primary and secondary antibodies used within the study. Antibodies were stripped between round 1 and 2.

Primary Antibody	Secondary antibody	Wavelength
Neurofilament DG Sensor (NFL; MCA-6H63; 1:4000, Biosensis, cat# M-2122-50; RRID: AB_2923484)	Goat Anti-mouse Alexa fluor 555 (Thermo Fisher, Cat# A-21422, RRID: AB_2535844)	Round 1 555 nm
IBA-1 (Ionized calcium-binding adapter molecule 1; 1:2000, Wako cat# 019–19741, RRID: AB_839504)	Goat Anti-rabbit Alexa fluor 647 (Thermo Fisher, Cat# A-21244, RRID: AB_2535812)	Round 1 647 nm
APP (amyloid precursor protein; 1:2000, Thermo Fisher, cat# 51–2700, RRID: AB_2533902)	Goat Anti-rabbit Alexa fluor 647 (Thermo Fisher, Cat# A-21244, RRID: AB_2535812)	Round 2 647 nm
Tomato Lectin (Lycopersicon esculentum; 1:1000, Vector Laboratories cat# B-1175, RRID: AB_2315475)	Streptavidin Alexa fluor 488 conjugate (Thermo Fisher, Cat# S11223, No RRID)	Round 2 488 nm

### Tissue analysis

For analysis, regions of interest were segmented, and a pixel classifier was used to detect positive staining. The classifier used Gaussian and Laplacian filters at a scale of 1.0 and resolution of 0.16 μm/pixel, with a minimum size of 3 μm for APP and NFL and 24 μm for IBA1, creating individual detection objects. All sections were then manually checked, and supplementary counts were added where positive staining was missed^53^. Furthermore, tomato lectin was used as an endothelial marker to evaluate non-specific vascular staining. Any co-localisation between the markers of interest and tomato lectin led to the removal of that detection object. A subset of slides was independently assessed by a second blinded reviewer, with high inter-rater reliability (> 0.9).

The spatial distribution of staining was also shown with QuPath’s ‘generate density map’ function which generates graded colour maps in accordance with density of detections. Density is defined as the number of detections in a 300 μm radius. This is colour coded, with black showing no nearby detections, and yellow showing the maximum detections.

### Serum analysis

A subset of ferrets underwent serum NFL and GFAP analysis, as selected by a random number generator. Analysis was conducted at Monash University by the Monash trauma group in the same manner described previously^58^, utilising the Simoa HD-X Analyzer (Quanterix, Billerica, MA, USA) with commercially available NFL or GFAP assays (V2; Cat No# 104073; Quanterix, Billerica, MA, USA). For GFAP analysis samples were examined from the 30 min (sham n/8, TBI n/15), 24 h (sham n/5, TBI n/6) and 72 h (n/8 group) timepoints, whilst for NFL only the terminal 24 h and 72 h samples were used (n/4 for shams, n/5 for injured ferrets). All protocols followed the manufacturer’s instructions. Samples were run in duplicate, and researchers were blinded to experimental groups.

### Statistical analysis

No ferrets were excluded. Data was graphed using GraphPad prism (v10.0.0). Data from all stains was averaged bilaterally and standardised to the region of interest area to create detections per mm^2^. ROI data was analysed using a two-way multivariate analysis of variance (MANOVA), with time and injury as the two factors in SPSS (v26). Sex was not considered as a factor, as the study was not powered to investigate this effect. Tukey’s corrections were applied for each region of interest. As it was anticipated that as few regions of interest would show injury, comparisons between ROI’s were not considered. For behaviour and serum analyses, t-tests, one or two-way ANOVAs with Bonferroni’s post hoc tests used as appropriate. Due to the limited sample size, ICP was analysed using a two-tailed Mann-Whitney U test and presented as median (IQR). Statistical significance was deemed when the p-value was less than 0.05.

## Results

One ferret in the 24 h survival timepoint was injured at 22 J, as this was the impact force used in previous adult studies^52^. Unfortunately, this ferret died immediately post-injury due to extensive skull fracture. Subsequently the impact speed was decreased to 17 J for all other injured ferrets, with CHIMERA reported impact energies comparable between timepoints (24 h: 13.20 [12.96–13.39]; 72 h: 13.28 [13.12–13.39]). At the 17 J impact speed *n* = 1 ferret died immediately post-injury in the 24 h cohort (8%) and *n* = 3 (all male) in the 72 h cohort (13%). This was associated with extensive skull fracture, that was typically diastatic in nature along the longitudinal suture around the injury site and associated with extensive blood loss.

**Fig. 3:**
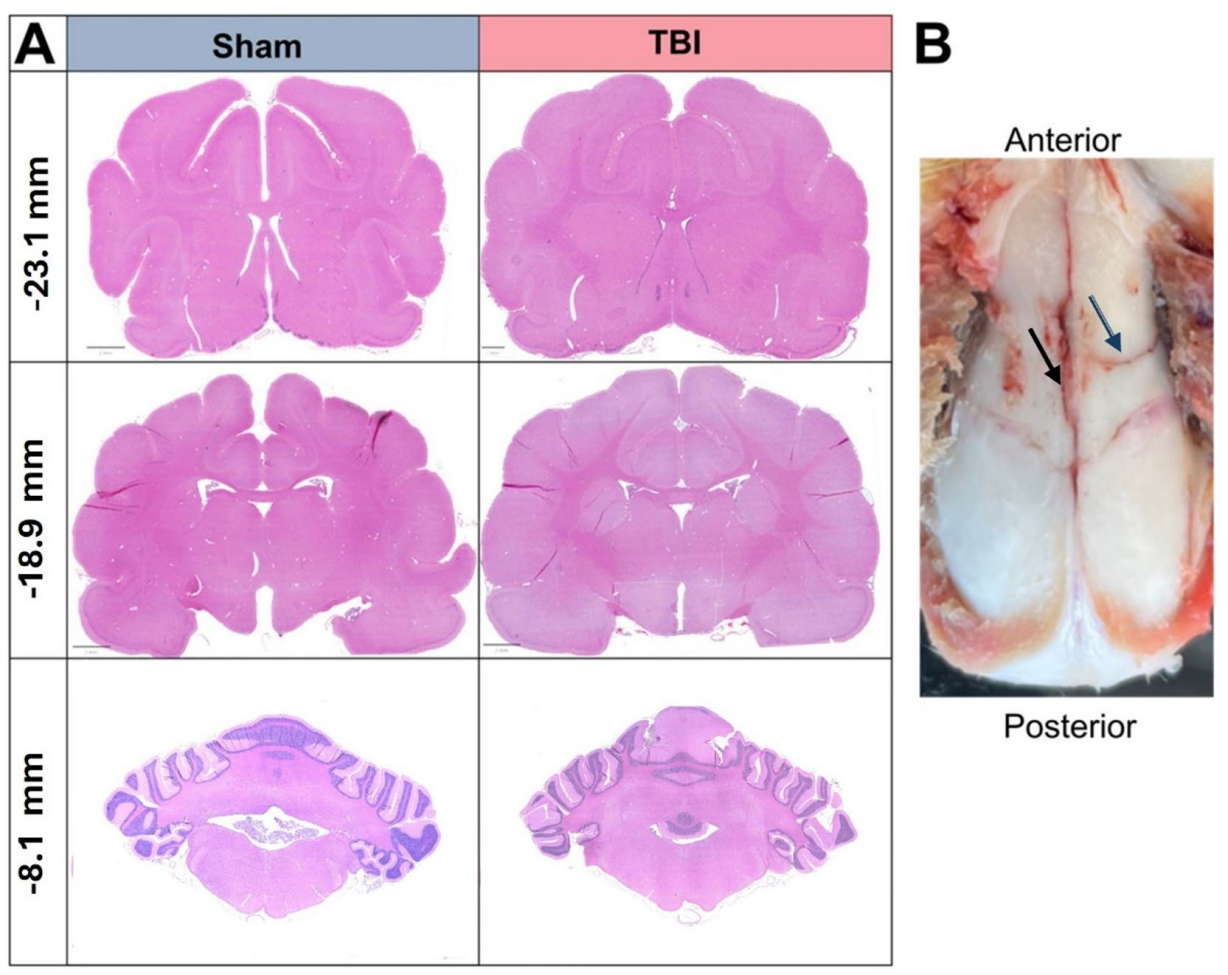
Macroscopic analysis. H&E staining across three antero-posterior levels in both sham and TBI animals show no evidence of contusions or haemorrhaging (**A**). Evidence of skull fracturing was observed in 50% of TBI animals, with most being diastatic. Here a fracture can be observed in sagittal suture (blue arrow) and coronal sutures (black arrow) (**B**).

Gross morphological examination of the brain found no notable focal contusions, confirmed with H&E with TBI animals appearing indistinguishable from sham animals (Fig. 3A). Of the animals that survived injury, 9/17 (53%) of animals also had minor diastatic skull fracture, that was evident at time of perfusion (Fig. 3B).

### Physiological parameters

HR and SpO2 nwere measured in the 15 min before and then to 15 min after injury and are presented as change compared to pre-injury baseline in Table 3. No interactions or main effects were noted for SpO2, with an overall effect of time-post-injury/sham seen for HR, with HR decreasing over-time with increasing exposure to isoflurane irrespective of group (F_1.4,33.6_ = 17.08; *p* < 0.0001).

**Table 3:** Physiological parameters to 15 min post-injury showing no differences between experimental groups, with HR decreasing over time in all ferrets. Differences between Sham and injured groups were analysed via two-way ANOVA. Values are reported as mean ± SD.

			Post-Injury		
			0–5 min	5–10 min	10–15 min
∆HR(BPM)	24 h	Sham	−9.8 ± 16.1	−13.0 ± 6.7	−14 ± 11.53
		TBI	2.2 ± 13.0	−6.8 ± 8.6	−13.6 ± 12.9
	72 h	Sham	−4.9 ± 7.78	−21.9 ± 18.6	−25.7 ± 20.9
		TBI	−1.1 ± 10.46	−16.2 ± 13.3	−28.8 ± 14.8
∆ SpO_2_ (%)	24 h	Sham	−1.5 ± 2	−1.8 ± 2.5	−1.0 ± 3
		TBI	−2.8 ± 2	−4.7 ± 6.0	−3.5 ± 1.7
	72 h	Sham	−1.0 ± 1.8	−0.6 ± 2.1	−0.2 ± 1.7
		TBI	−2.0 ± 2.4	−1.3 ± 3.2	−1.6 ± 3.3

### Intracranial pressure

There was no difference in ICP with injury (Sham: 3.56 (IQR 2.40–5.17) vs. TBI: 5.51 (IQR 3.64–17.23), U = 4, *p* = 0.34), with no significant effect of injury on any other physiological parameter at 24 h (Table 4).

**Table 4:** Physiological parameters during ICP monitoring. Differences between Sham and injured groups were analysed using a two-tailed Mann–Whitney U test. Data are presented as median (IQR); *n* = 4 per group.

	Sham	TBI	*p* value
Mean arterial pressure (mmHg)	53 (IQR 48.3–67.5)	55.5 (IQR 37.5–96.0)	0.34
HR (BPM)	229.5 (IQR 226.8–233.8.8.8)	234.0 (IQR 199.0–259.3.0.3)	> 0.99
SpO_2_ (%)	100 (IQR 100–100)	97 (IQR 93.25–100)	0.43
Arterial pO_2_ (mmHg)	88.5 (IQR 81.25–93.5)	74.5 (IQR 116-66.75.75)	0.37
Venous pO_2_ (mmHg)	66.5 (IQR 61–72)	53.5 (IQR 44.8–62.3)	0.11
Arterial pCO_2_ (mmHg)	109.7 (IQR 129.4–88.8)	96.3 (IQR 84–106.1.1)	0.34
Venous pCO2 (mmHg)	106.3 (IQR 94.1–126.7.1.7)	102.6 (IQR 91.8–106.7.8.7)	0.40

### Functional outcome

#### Motor outcome

**Fig. 4:**
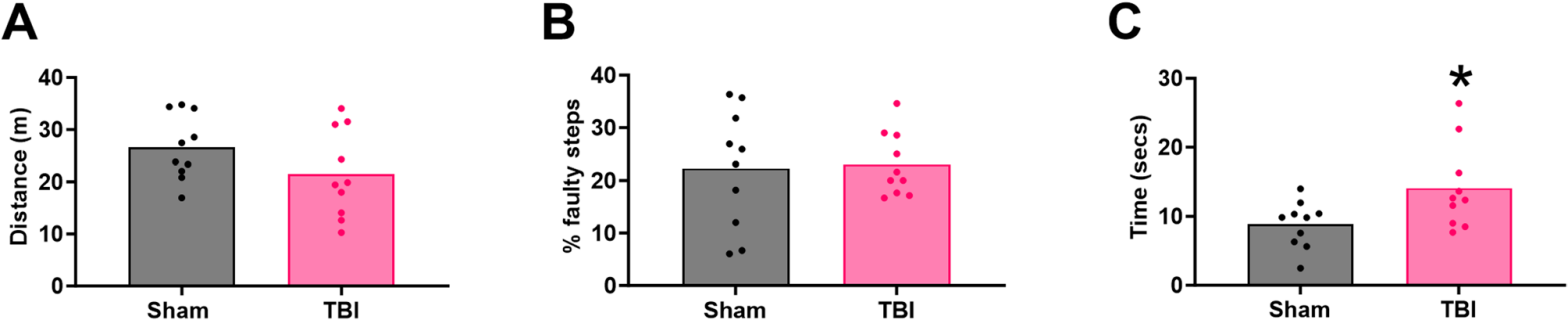
Motor outcome. Distance travelled on the open field was not significantly different between sham and injured ferrets (**A**). Examination on performance on the more demanding ladder task, found no difference in % faulty steps (**B**), but an increase in time to cross with injury (C). Data was analysed using two-tailed t-tests; *p* < 0.05. Values are presented as mean ± SEM; *n* = 10 per group.

The open field was used to examine general locomotor activity at 1 d post-injury (Fig. 4A), and the ladder for balance and coordination at 2 d post-injury (Fig. 4B-C). There was no significant difference in distance travelled in the open field following injury compared to shams (21.52 ± 8.39 vs. 26.62 ± 6.27 m, t_9,9_=1.79, *p* = 0.14). On the ladder walk, the percentage of faulty steps was comparable between sham and injured ferrets (22.27 ± 11.23 vs. 23.03 ± 6.05%, t_9,9_=3.44, *p* = 0.85), although injured ferrets took significantly longer to cross the ladder (14.08 ± 6.12 v 8.85 ± 3.37s, t_9,9_=3.44, *p* < 0.05).

### Cognitive outcome

**Fig. 5:**
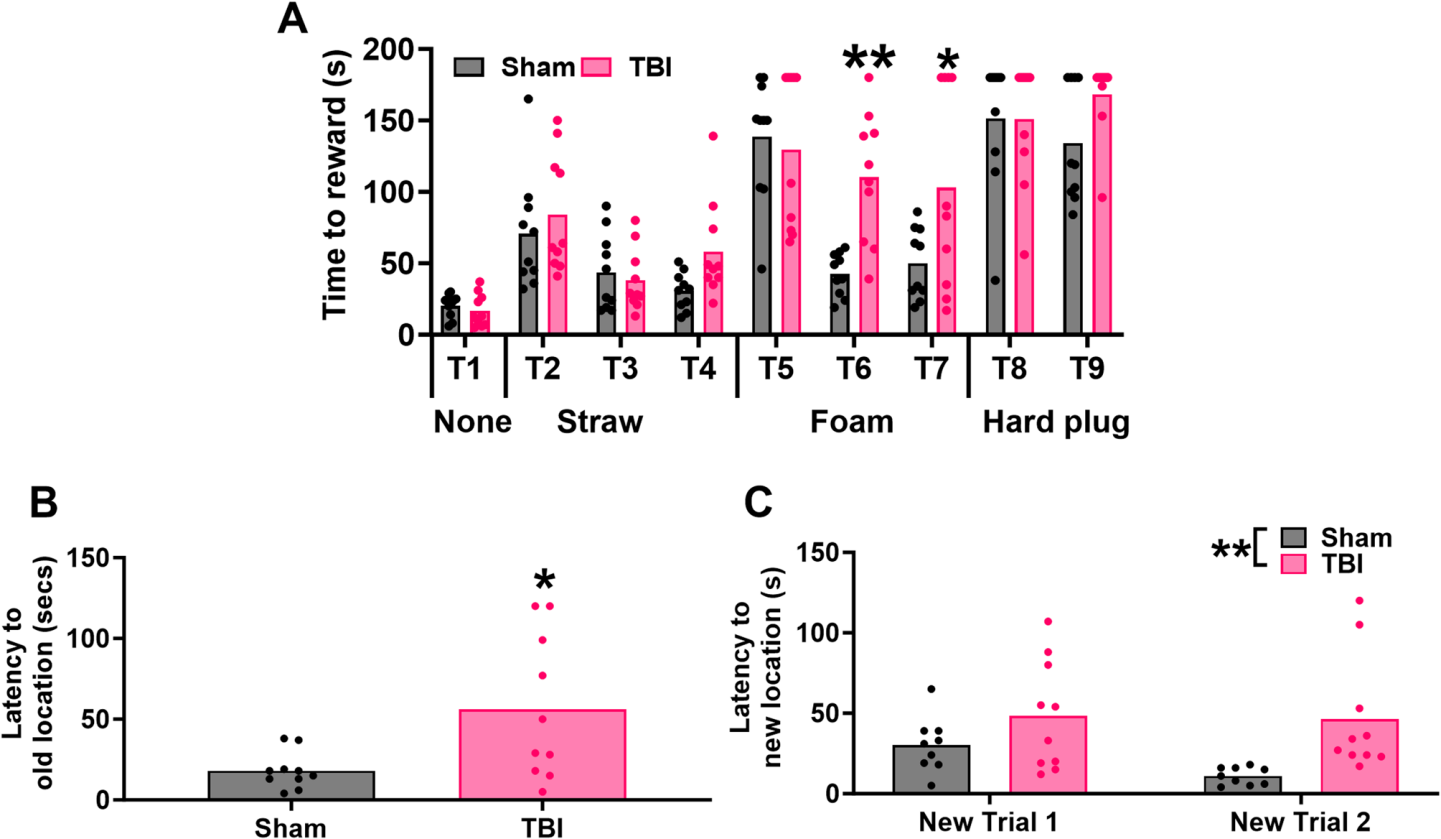
Cognitive outcome post-injury was assessed on the puzzlebox (**A**) and quadrant maze (B-C). Injured ferrets exhibited significantly increased latency to reward on trials 5 and 6 of the puzzle box, increased latency to a previously learned location in the quadrant maze (**B**), and impaired acquisition of a new location in the quadrant maze (**C**). Data was analysed using two-way ANOVA (A, C) or two-tailed t-tests (B); **p < 0.01, *p < 0.05. Values are presented as mean; *n* = 10 per group.

Cognition was tested on the puzzlebox (Fig. 5A) which evaluates executive function, long and short-term memory and on the Quadrant Maze which examines spatial memory and cognitive flexibility (Fig. 5B-C). On the puzzle box, there was a significant interaction between injury and trial number (F_8,144_=3.19; *p* < 0.01). Post-hoc analyses found no significant difference on any of the problem-solving trials (TBI vs. sham: T1: 84.3 ± 41.42 vs. 70.70 ± 39.96, *p* = 0.99; T4: 129.6 ± 54.21 vs. 138.60 ± 42.70, *p* = 0.99; T8: 150.90 ± 26.78 vs. 151.60 ± 46.83, *p* > 0.99). Similarly, there was no effect of injury in the short or long-term memory trials for the easiest obstruction, straw (T2,3), nor the hardest obstruction, wooden-plug (T9). However, there were significant differences for the foam plug for both the short- and long-term memory trials, with injury increasing latency to reward (TBI vs. Sham: T6:110.30 ± 45.10 vs. 42.50 ± 14.93s, *p* < 0.01); T7: 103.00 ± 70.11 vs. 49.90 ± 24.73s, *p* < 0.05).

In the quadrant maze, injured ferrets took longer to enter the reward location that had been learnt prior to injury (56.10 ± 44.42 vs. ± 18.10 ± 11.26s, t_9,9_=2.62, *p* < 0.05) and were impaired in their ability over two subsequent trials to learn a new location for the reward. There was a main effect of injury (F_1,17_=7.17; *p* < 0.05), but no interaction between injury or trial (F_1,17_=1.34; *p* = 0.26), nor main effect of trial (F_1,17_=2.04; *p* = 0.17).

### Histological evaluation

#### Axonal injury

**Fig. 6:**
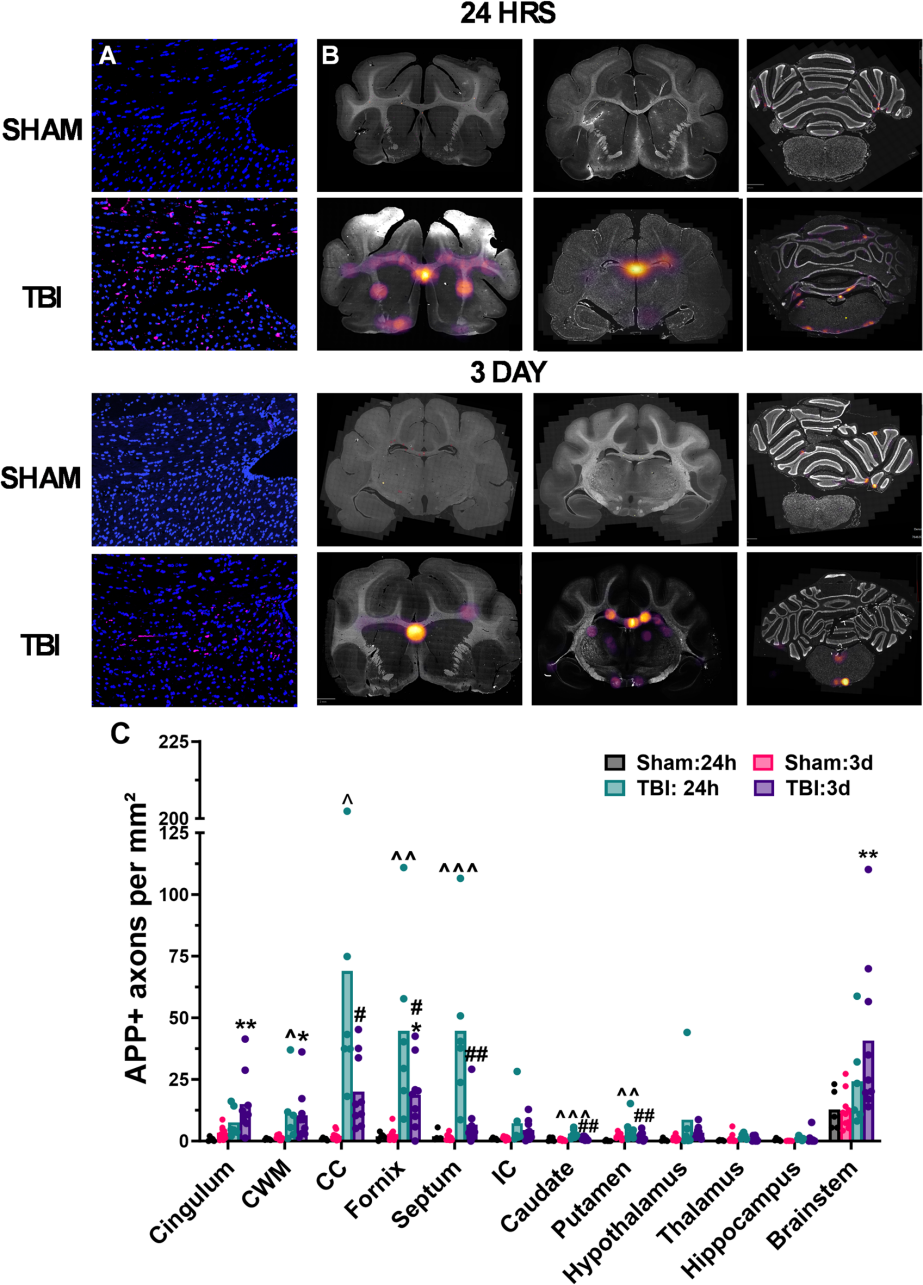
Analysis for APP + axons in key white and grey matter regions. Representative images (**A**) of APP immunostaining (pink) at the border of the corpus callosum and fornix show that injury led to the typical accumulation of APP within axons creating swollen bulbs and lengths. Generation of heat-maps (**B**) demonstrated that APP + axonal injury was concentrated in the midline and was more prominent at 24 h than 72 h. Quantitative analysis found a significant interaction of time and injury, with increases in APP + axonal injury at 24 h within key white matter tracts including the cortical white matter, corpus callosum, fornix and septum and in the grey matter structures the caudate and putamen (**C**). APP + axonal injury decreased by 72 h post-injury, with significant increases only found in the cingulum, cortical white matter, fornix and brainstem. Data was analysed using two-way MANOVA, with Tukey’s corrections for each region of interest. ^*p* < 0.05, ^^*p* < 0.01, ^^^*p* < 0.001 vs. 24 h sham, *p < 0.05, **p < 0.01 vs. 72 h sham, #*p* < 0.05, ##*p* < 0.01 vs. 24 h TBI, Graphs are presented as mean. *n* = 5–6 per group for 24 h ferrets, *n* = 10 per group for 3 day-ferrets.

Axonal injury was analysed with two markers, APP for transport disruption, and for neurofilament (NF) pathology, with the injury-specific NFL marker, MCA-6H63. Analysis of APP immunostaining revealed numerous APP + swollen lengths within white matter tracts like the corpus callosum and punctate dots of the transverse axons in the fornix following injury, with minimal staining in sham ferrets (Fig. 6A). Density maps of APP + axonal pathology were generated, revealing hot spots in the midline, particularly in the corpus callosum and fornix, with this most prominent at 24 h following injury (Fig. 6B). Across the three sections examined, APP + axonal pathology was most evident in the most anterior − 23.1 mm section.

A two-way MANOVA on the number of APP + axons within each of the brain regions of interest, found a significant interaction of injury x time (Pillai’s Trace = 0.73 F_12.14_=3.30, *p* < 0.01, ηp^2^ = 0.77), driven by significant differences in the cingulum (*p* < 0.01), cortical white matter (*p* < 0.05), corpus callosum (*p* < 0.001) fornix (*p* < 0.001), septum (*p* < 0.001), caudate (*p* < 0.001), putamen (*p* < 0.05) and brainstem (*p* < 0.05, Fig. 6C). Post-hoc analyses showed significant increases relative to shams following injury at 24 h in the corpus callosum (68.92 ± 67.94 vs. 0.83 ± 0.45 APP + axons/mm^2^, *p* < 0.05), fornix (44.63 ± 36.55 vs. 1.96 ± 1.40 APP + axons/mm^2,^ *p* < 0.01) and septum (44.67 ± 33.66 vs. 1.94 ± 20.5 APP + axons/mm^2^*p* < 0.001), with more modest but still significant increases in the caudate (2.93 ± 1.85 vs. 0.64 ± 0.77 APP + axons/mm^2,^ *p* < 0.001), putamen (5.03 ± 5.32 vs. 0.11 ± 0.25 APP + axons/mm^2,^ *p* < 0.01) and cortical white matter (11.40 ± 13.22 vs. 0.71 ± 0.26 APP + axons/mm^2^*p* < 0.05). By 72 h post-injury, APP + axons had decreased in all regions except the brainstem and cingulum, which had increased and were now significantly different relative to shams (Brainstem: 40.78 ± 32.35 vs. 12.77 ± 8.28 APP + axons/mm^2,^ *p* < 0.01; Cingulum: 14.99 ± 12.39 vs. 3.30 ± 2.73 APP + axons/mm^2,^ *p* < 0.01). The only two other regions that remained significantly elevated compared to shams at 72 h post-injury were the cortical white matter (10.52 ± 10.78 vs. 1.43 ± 0.82 APP + axons/mm^2^, *p* < 0.05) and fornix (18.73 ± 13.83 vs. 2.79 ± 2.61 APP + axons/mm^2^, *p* < 0.05) Indeed, levels of APP + axonal injury significantly decreased from 24 h to 72 h post-injury in the corpus callosum (19.98 ± 14.88 APP + axons/mm^2^, *p* < 0.01), fornix (*p* < 0.05), septum (6.69 ± 8.85 APP + axons/mm^2^, *p* < 0.01), caudate (0.99 ± 0.64 APP + axons/mm^2^, *p* < 0.01) and putamen (1.88 ± 1.68 APP + axons/mm^2^, *p* < 0.05).

**Fig. 7:**
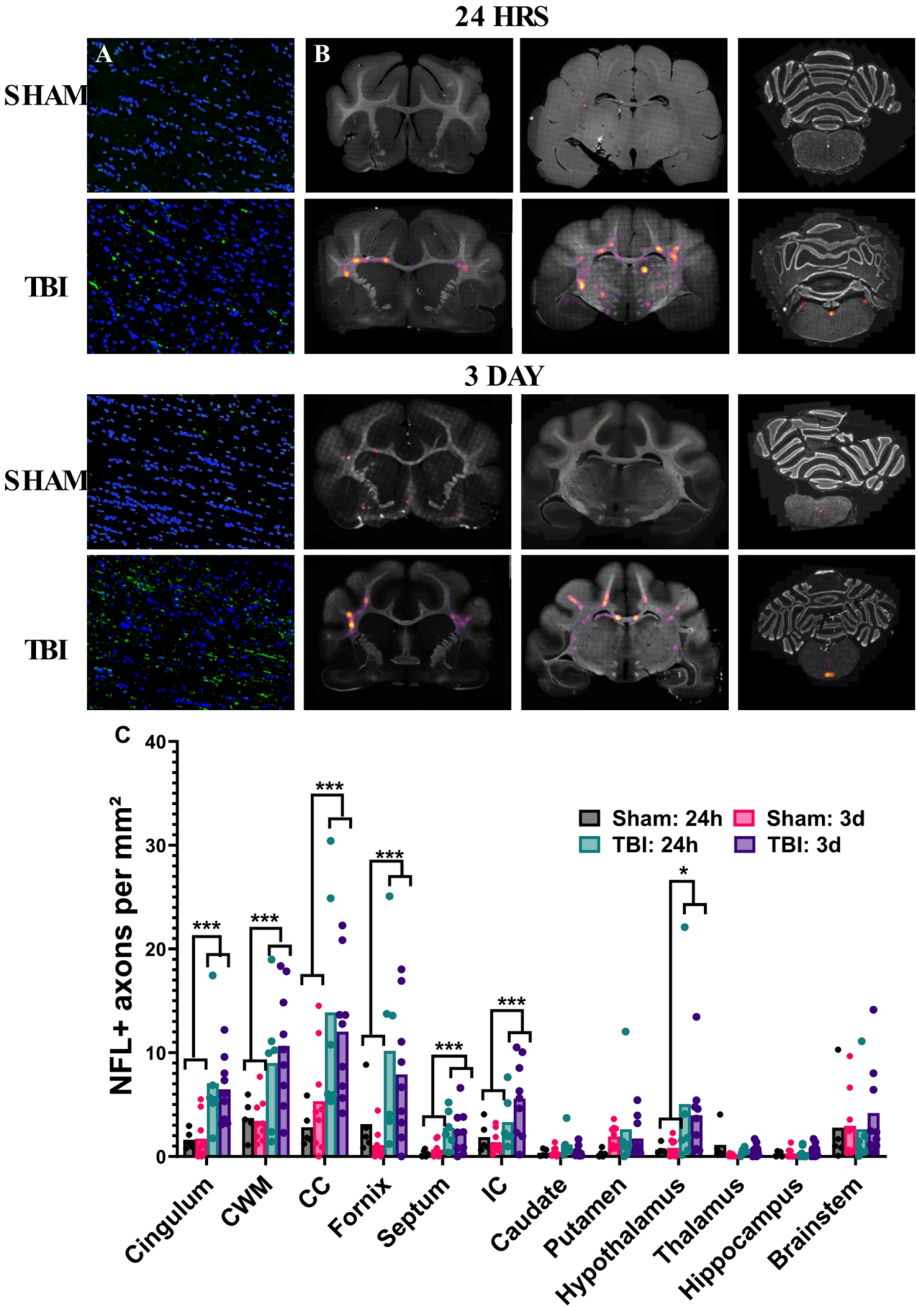
Analysis of NFL + axons following injury in key white and grey matter regions. Representative images taken at the border of the cortical white matter and corpus callosum show distinctive swollen axonal lengths and bulbs (green) (**A**), with heat-maps demonstrating that NFL + axonal injury was prominent in the lateral cortical white matter and corpus callosum, as well as within central structures like the fornix (**B**). Quantitative analysis found that overall injury increased NFL + axons within these white matter regions, as well as the hypothalamus, with no difference between the 24 h and 72 h timepoint (**C**). Data was analysed using two-way MANOVA, with Tukey’s corrections for each region of interest. *p < 0.05, ***p < 0.001. Graphs are presented as mean. *n* = 5–6 per group for 24 h ferrets, *n* = 10 per group for 3 day-ferrets.

Like APP + staining, NFL also identified many undulated axons, with numerous axonal bulbs, although these were concentrated more laterally, with a predilection for the cortical white matter/corpus callosum border (Fig. 7A). Generation of the heat-maps demonstrated that NFL axonal pathology following injury was particularly evident in the cortical white matter, cingulum, fornix and corpus callosum, with similar staining in the two cortical sections, and minimal injury specific staining in the brainstem (Fig. 7B). Quantitative analysisrevealed no significant interaction of injury x time (Pillai’s Trace = 0.55; F_12,14_=1.41, *p* = 0.37), but a main effect of injury (Pillai’s Trace = 0.81; F_12,14_=5.00, *p* < 0.01, ηp^2^ = 0.81), but not time (Pillai’s Trace = 0.53; F_12,14_=1.32, *p* = 0.37). Injury significantly increased NFL + axons within the cingulum (6.81 ± 4.00 vs. 1.68 ± 1.73 NFL + axons/mm^2^, *p* < 0.01), cortical white matter (10.00 ± 5.78 vs. 3.53 ± 1.93 73 NFL + axons/mm^2^, *p* < 0.01), corpus callosum (12.79 ± 8.22 vs. 4.42 ± 4.25 NFL + axons/mm^2^, *p* < 0.01), fornix (8.82 ± 7.36 vs. 1.82 ± 2.35 NFL + axons/mm^2^, *p* < 0.01), septum (2.48 ± 1.95 vs. 0.53 ± 0.61 NFL + axons/mm^2^, *p* < 0.01), internal capsule (3.16 ± 2.97 vs. 1.55 ± 1.18 NFL + axons/mm^2^, *p* < 0.01) and hypothalamus (4.41 ± 5.93 vs. 0.78 ± 0.70 NFL + axons/mm^2^, *p* < 0.05, Fig. 7C).

### Microglial response

**Fig. 8:**
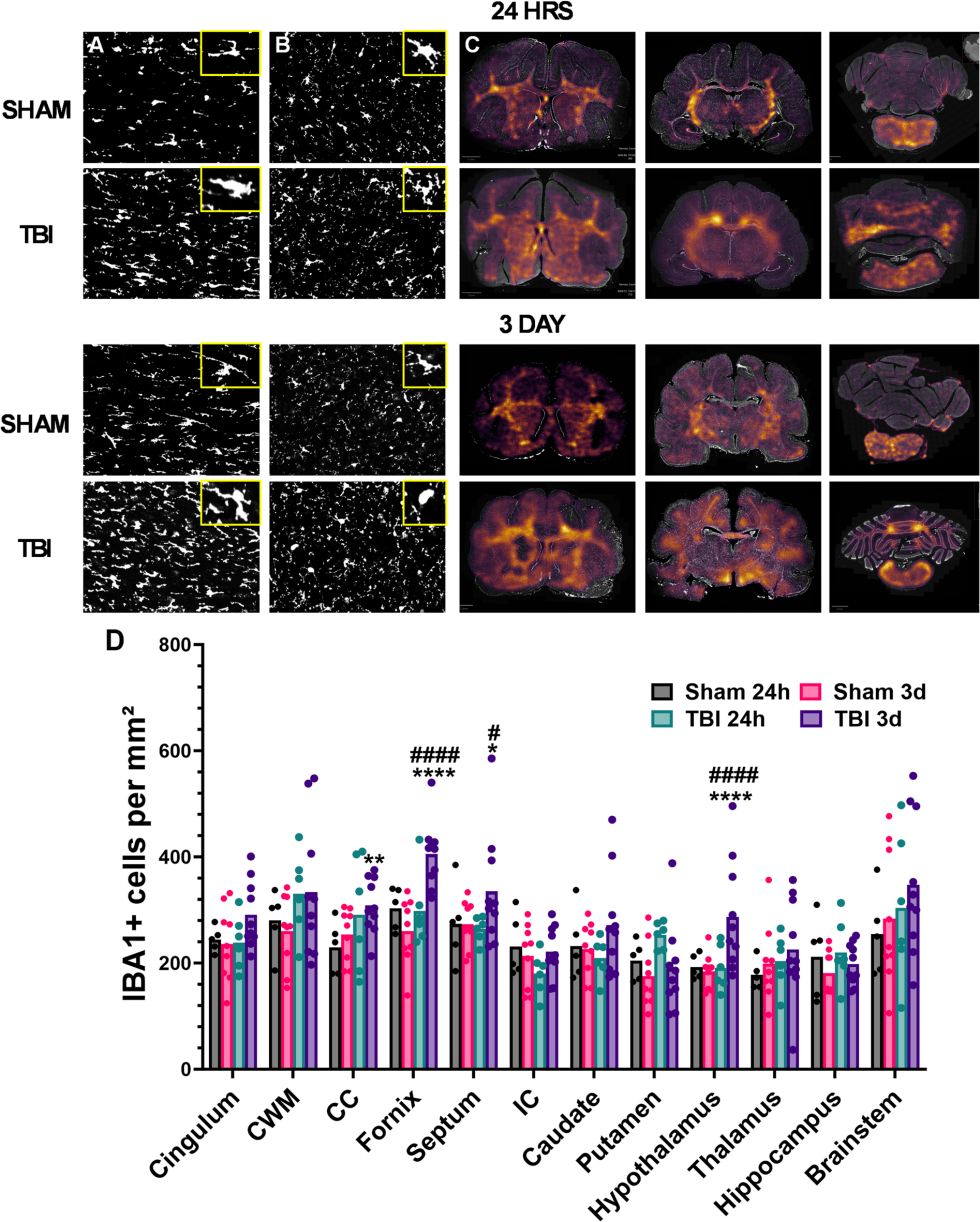
Analysis for IBA-1-positive cells in key white and grey matter regions following injury. Representative images from the corpus callosum (**A**) and hypothalamus (**B**) with insets to show typical morphology, with thickening and shortening of microglial branches with injury following creation of binary images from thresholding. Generation of heat-maps shows that IBA1 + density was seen particularly within the white matter and regions directly under the impact (**C**). Quantitative analysis (**D**) shows significant increases at 72 h post-injury in the corpus callosum, fornix, septum and hypothalamus. Data was analysed using two-way MANOVA, with Tukey’s corrections for each region of interest. ****p < 0.001, *p < 0.05 compared to 72 h shams, ####*p* < 0.001, #*p* < 0.05 compared to 24 h TBI ferrets. Graphs are presented as mean. *n* = 5–6 per group for 24 h ferrets, *n* = 10 per group for 3 day-ferrets.

The effect of injury on microglia was examined via IBA1 staining. At 24 h post-injury minimal changes in morphology within either white (Fig. 8A) or grey matter (Fig. 8B) were noted, with some thickening of branches and retraction seen. By 72 h post-injury an overall increase in the number of microglia was evident in the white matter, with microglia demonstrating changes in morphology including smaller thicker branches and an increase in soma size. Generation of heat maps shows this overall increase in microglia number at 72 h, particularly within central white matter structures (Fig. 8C).

A two-way MANOVA of the number of IBA1 + cells within each of the brain regions of interest, with injury and time the between group factors was conducted. There was a significant interaction of injury x time (Pillai’s Trace = 0.72; F_15,12_=3.30, *p* < 0.05, ηp^2^ = 0.72), driven by significant differences in the corpus callosum (*p* < 0.05) fornix (*p* < 0.001), septum (*p* < 0.05) and hypothalamus (*p* < 0.001, Fig. 8D). Post-hoc analyses found that IBA1 + cells increased at 72 h relative to their shams in each of these regions (Fornix: 445.08 ± 03.87 vs. 260.64 ± 62.52 IBA1 + cells/mm^2^, *p* < 0.001; Corpus callosum: 349.88 ± 95.40 vs. 245.23 ± 45.02 IBA1 + cells/mm^2^, *p* < 0.01; Septum: 348.65 ± 96.16 vs. 273.44 ± 44.49 IBA1 + cells/mm^2^ *p* < 0.05; Hypothalamus: 314.26 ± 96.08 vs. 184.04 ± 33.39 IBA1 + cells/mm^2^*p* < 0.001) (Fig. 7C). This effect was not seen at 24 h, with no significant differences relative to their shams (Fornix: 298.50 ± 70.05 vs. 303.00 ± 39.58 IBA1 + cells/mm^2^, *p* = 0.92; Corpus callosum: 290.83 ± 49.83 vs. 229.83 ± 49.83 IBA1 + cells/mm^2^, *p* = 0.22; Septum: 264.17 ± 23.59 vs. 253.50 ± 43.57 IBA1 + cells/mm^2^ *p* = 0.79; Hypothalamus:, 190.83 ± 42.65 vs. 192.50 ± 23.52 IBA1 + cells/mm^2^, *p* = 0.97), such that the 24 h injured ferrets had significantly less IBA1 + cells than 72 h injured ferrets in the fornix (*p* < 0.001), internal capsule (*p* < 0.05) and hypothalamus (*p* < 0.001).

### Serum biomarkers

**Fig. 9:**
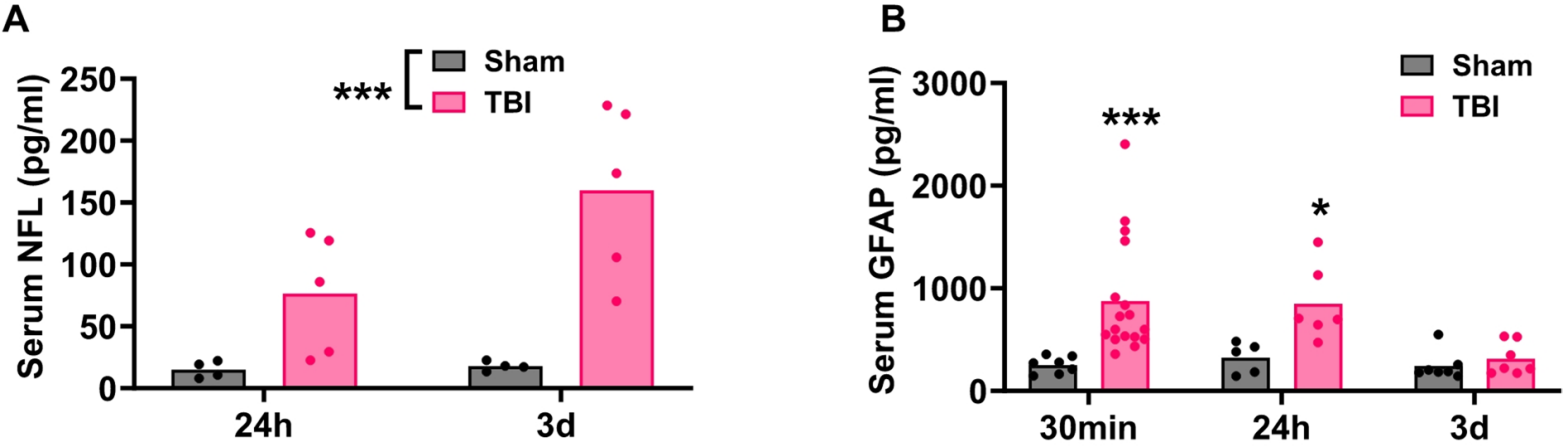
Serum NFL (**A**) and GFAP (**B**) following injury Data were analysed using two-way ANOVA. **p < 0.001, *p* < 0.05 compared with sham ferrets. Graphs are presented as mean. NF-L: *n* = 4–6 per group. GFAP: 30 min: sham *n* = 8, TBI *n* = 15; 24 h: sham *n* = 5, TBI *n* = 6; 72 h: *n* = 8 per group.

Serum NFL levels were examined at 24 h and 72 h post-injury, and serum GFAP levels at 30 min, 24 h and 72 h. For NFL there was no significant interaction (F_1,18_=3.30 = 0.48, *p* = 0.50), but a main effect of injury (F_1,18_ =5.70, *p* < 0.05), with injury leading to a substantial increase in NFL (228.2 ± 89.92 vs. 15.72 ± 2.81 pg/ml; Fig. 9A). In contrast a significant interaction of time x injury was found for GFAP serum levels (F_2,41_=3.11, *p* < 0.05), with injury causing significant elevations at 30 min (894.27 ± 575.58 vs. 252.37 ± 81.83pg/ml, *p* < 0.001) and 24 h (848.98 ± 365.03 vs. 323.51 ± 150.41pg/ml, *p* < 0.05) but not at 72 h post-injury (278.5 ± 142.31 vs. 243.36 ± 138.77pg/ml, *p* = 0.95) (Fig. 9B).

## Discussion

This is the first description of paediatric ferret model of TBI, where we have successfully generated a diffuse axonal injury response, without focal contusions in 2–3-month-old ferrets, equivalent to injury in ~ 3–5-year-olds clinically. Axonal injury was detected via two makers, APP for transport disruption and NFL for neurofilament damage. Axonal transport disruption peaked at 24 h and was particularly localised in midline periventricular structures such as the corpus callosum and fornix. NFL + axonal pathology persisted to 72 h post-injury, in line with elevated serum NFL levels out to this timepoint, with a more lateralised pattern of involvement, particularly in the cortical white matter. An increase in microglia was evident by 72 h post-injury, confined to the white matter tracts with the highest amounts of axonal injury, and in the hypothalamus. Injury led to subtle motor deficits, detected on the ladder walk, and cognitive deficits in short- and long-term memory and cognitive flexibility on the puzzle box and quadrant maze.

Initially the same impact force previously reported to induce mild injury in adult ferrets was applied^52^, but this resulted in extensive skull fracture in the younger animal. Younger age is associated with a thinner more compliant skull, that undergoes greater elastic deformation^19, 59^, predisposing to fracture at lower energies. Consistent with this, we observed skull fracture in ~ 50% of our cohort even with the lower 17 J impact compared to 7.5% at 22 J in adults. This pattern mirrors clinical observations of a higher incidence of skull fracture in paediatric compared with adult populations^60, 61^. Although the fracture rate observed here (~ 50%) exceeds commonly reported clinical estimates of ~ 15–20%^62, 63^, skull fractures, particularly diastatic fractures, may be under-recognised in young children. Incomplete sutural ossification limits the sensitivity of standard skull radiographs^64^ and although CT scanning is more sensitive for fracture detection, it is performed in only 20^65^−50%^66^ of paediatric TBI presentations. Notably, in a large US emergency department cohort of children under four, in those who underwent head CT, skull fractures were observed in 53% of cases^67^. To further evaluate the translational validity of this ferret model, future studies incorporating detailed biomechanical analyses and direct comparison with clinically relevant paediatric head injury metrics are warranted.

Nonetheless, at the reduced 17 J impact our overall mortality of 11% was comparable to the 7.5% mortality reported at the higher impact speed in adults, which is supported by previous preclinical studies that alteration to injury parameters is required in paediatric models. Indeed, using the same impact parameters in the fluid percussion model resulted in higher mortality in p17 vs. adult rats, with a 100 vs. 55% mortality rate for a 3.65 atm injury^29^. Furthermore, a weight drop with a 100 g weight produced similar injuries in a p17 rat compared to adults injured with a 450 g weight^21^. These alterations relate not just to the decreased size in paediatric animals, but also differences in the properties of the brain itself, with the immature brain being stiffer than the adult brain due to the smaller amounts of myelin and hence higher brain water content^37^, leading to greater strains within the tissue when the same force is applied which may differentially affect the response of axons to injury^68^.

Axons consist of a core of microtubules to facilitate axonal transport, which are surrounded by neurofilaments that act as space filling polymers^69^. Neurofilaments contain three isoforms, designated as light (NFL; 68 kDa), medium (NFM; 168 kDa) and heavy (NFH; 200 kDa). The more abundant NFL forms the backbone, with NFM and NFH located more peripherally, as their longer tails form sidearms that protrude from the filament core^70^. Here, axonal Injury evaluated by accumulation of axonally-transported APP, was 2-3x higher at 24 h than previously reported with injury at 22 J in adults, but had decreased to similar levels by 72h^52^. Nonetheless, the distribution of this APP + axonal injury was similar to adults with a predilection for midline periventricular structures including the fornix, septum and corpus callosum. Indeed, this distribution of axonal injury has previously been reported in rodent models of diffuse TBI in rodents of a similar equivalent age, also peaking at 24 h, although none of these studies specifically quantified axonal injury burden relative to shams^26, 27, 37, 71^. The proximity of these structures to the ventricles generate additional shearing forces with rotational acceleration due to the differences in tissue density between cerebrospinal fluid and tissue^72^. Microtubules, as the stiffest axonal component, appear to be most vulnerable to this mechanical stretch, with dynamic loading of axons leading to rapid rupture^73^, with subsequent accumulation of axonally-transported proteins. Of note a more rapid resolution of APP + axonal injury was observed in periventricular structures in this paediatric model than seen in adults, with a return to adult like levels of APP + axonal injury by 72 h. Fast axonal transport rates are quicker in younger animals^74^ which may permit APP to rapidly accumulate, but may in turn also lead to rapid resolution of the microtubules affected by mechanical disruption that could be repaired to restore axonal transport. Following injury, at least in adults, a subset of axons increase expression of microtubule stabilisation proteins associated with repair, like MAP6^75^, with not all axons with APP pathology going on to degenerate^76^. Further work is needed to determine if this axonal stabilisation response differs across the lifespan.

In contrast to APP + axonal pathology there was much less neurofilament axonal pathology, as detected with the injury specific MCA-6H63 NFL antibody^77^. The MC1-6H63 antibody belongs to the DG sensor antibody series that were developed to recognise epitopes within the Coil 2 region NFL, like the UMAN type antibodies used in NFL ELISA kits^77^. This epitope becomes exposed only after axonal injury, enabling degeneration-specific detection^77, 78, 79^. Consistent with this, MCA-6H63 staining has been assessed in spinal cord injury models, where it selectively labels degenerating axons and co-localises with established markers of axonal injury, such as APP, while remaining absent in uninjured controls^78^. Here MCA-6H63 NFL + axonal injury showed a more diffuse pattern than APP + staining, involving not just the corpus callosum and fornix, but also in the cortical white matter and hypothalamus. Following injury, NFM and NFH sidearms are either physically damaged^80^ or subject to the effects of enzymes activated by the unregulated influx of calcium, including calcineurin^80^ and calpain^81^, which dephosphorylate sidearms to promote compaction of neighbouring fibres and direct degrade neurofilaments. In paediatric pre-clinical models neurofilament pathology has been detected with antibodies directed towards NFH^71^ and NFM^26^ in rodents and NFL^37^ in piglets, with these antibodies similarly detecting less axonal injury than APP. In previous studies, neurofilament pathology was similarly widespread involving the corpus callosum, cingulum and cortical white matter^26, 71^, and appeared to affect different axons to those showing APP + axonal pathology, even within the same white matter tract^26^. This aligns with the different distribution of APP + and NFL injury seen here. Notably the amount of NFL + axons was ~ 2x less in the corpus callosum than previously reported following CHIMERA injury in adult ferrets^52^, which may simply reflects that children have less neurofilaments within axons than adults. Axons increase in radial size throughout the development due to the accumulation of neurofilaments, particularly in large myelinated axons like those in the corpus callosum^82^. Nonetheless, injury was still associated with an increase in serum NFL out to 72 h post-injury, in line with clinical work where NFL was elevated to 48 h in moderate-severe paediatric TBI patients, the last time-point assessed^83^, and from 24 h to at least one week following injury in toddler equivalent piglets^45^. This provides further evidence that the paediatric ferret brain injury model replicates key features of clinical head injury.

Alongside axonal injury, the response of microglia to the insult was examined, with microglia numbers increasing by 72 h post-injury in most injured white matter tracts, alongside the hypothalamus which would serve as a site of contra-coup injury^52^. In pre-clinical adult models of diffuse TBI injury is similarly associated with accumulation of monocytes and macrophages within injured white matter tracts^84–86^, with clinical cases showing more microglia in the white matter compared to grey matter^87^. To date, no other diffuse paediatric TBI models have investigated the microglial response in white matter. Following mFPI in age equivalent rats, only the grey matter was examined, with no change in IBA1 + number found in the cortex under the impact site^88^, hippocampus, hypothalamus or motor cortex^89^, with only subtle changes in branch length and process length in the perilesional cortex^88^. Further work is required to examine the transcriptomic and proteomic responses within these microglia in response to injury, to understand if it leads to functional changes. Furthermore, whether this microglial response could interact with neurodevelopmental processes where microglia are required to remove unwanted synapses requires further investigation^90^. The microglial response to injury has also been suggested to differ with age, with the suggestion that following diffuse TBI in younger mice, microglia play a protective role, with depletion increasing degenerating cells within white matter, with no effect on axonal injury^91^. It should also be noted that the inflammatory response to injury was associated with a rapid increase in serum GFAP levels, seen within 30 min, in line with clinical reports that GFAP is increased post-TBI^92–94^, but rapidly returns to baseline beyond 24 h in most children with mTBI^93^.

ICP was also monitored in a small cohort to confirm that the injury was not sufficient to lead to increases in ICP^95^. Clinically, raised ICP, typically defined as pressures above > 20mmHg, is associated with severe TBI, and even then is only evident in ~ 50% of paediatric head injury patients^96^. In other pre-clinical diffuse gyrencephalic models of toddler equivalent injury, extensive subdural, subarachnoid and intraparenchymal haemorrhage were required to produce raised ICP, suggesting the need for a mass effect. Indeed, thinner cranial bones, and less myelinated neural tissue may allow a greater accommodation of volume, before ICP becomes sustainably elevated^95^.

Supporting the mild-moderate nature of the injury, minor deficits in motor and cognitive performance were noted, with impaired balance on the ladder and short and term memory deficits in the puzzle box and quadrant maze. While these tests have been validated in adult ferrets, the ability to detect subtle behavioural impairments could be reduced by repeated exposure and the small sample sizes. Importantly these deficits are consistent with those reported following concussion in children. In a cohort of 303 children less than six years of age common early post-injury symptoms included balance difficulties, attention and concentration deficits and reduced processing speed^97^. The functional deficits observed here align with the distribution of axonal injury as reduced white matter integrity within the corpus callosum^98^ and pyramidal tract^98, 99^ correlated with poor gait and motor performance in paediatric TBI. Furthermore, the cingulum^100^, corpus callosum^101^ septum^102^ and fornix^103^ are all associated with cognitive functions including episodic memory, working memory and processing speed and are commonly injured in paediatric TBI^104–106^. Consistent with this, pre-clinical models of diffuse TBI in a toddler-age equivalent mice also demonstrated acute motor deficits in the ladder task and beam walking^107^. Only more severe injury models with ~ 50% mortality have assessed cognition acutely in rats, reporting deficits on the Morris Water Maze within the first week following injury^24, 33^. Indeed, a milder injury in toddler aged rats did not impair performance on the Morris Water Maze at 28 d post-injury^27^, but whether this reflects resolution of earlier deficits is not known. Future studies utilising this ferret model should therefore examine the trajectory of functional recovery beyond 72 h and its interaction with ongoing neurodevelopment, including whether deficits resolve, persist or emerge later during adolescence or adulthood. To date, behavioural outcomes following diffuse TBI in gyrencephalic toddler-preschool aged equivalent animals have not been conducted. Friess et al. used a non-impact impulsive loading model, but in 3−4d old infant equivalent piglets, demonstrating lower interest in exploring their environment and higher failure rates in visual-based problem solving compared to shams at 24 h post-injury^42^. However in older pigs, only the functional effects of focal injury have been investigated with 3–4 week old pigs, showing impaired spatial memory in a T maze and gait abnormalities^108^. Here we demonstrate that ferrets provide a viable gyrencephalic model alternative to pigs, and that the behavioural tasks previously only assessed in adults can also be reliably used in paediatric ferrets.

These results should be interpreted in the context that the 72 h cohort ferrets were slightly older and mixed sex compared to the all-male 24 h cohort, which may have led to differences in the injury response at 24 h compared to 72 h These changes were driven by difficulty in the availability of ferrets in Australia. The ferrets had a short breeding season and thus availability of paediatric ferrets was limited to a short window each year (~ 2–3 months). Nonetheless, previous work has shown that 3–5 day old pigs injured with the same parameters as 4 week old pigs had higher axonal burden at 6 h post-injury, even when alterations in brain mass and stiffness were taken into account^37^. However, in this case infant equivalent animals were being compared to toddler equivalent pigs, whereas our ferret cohorts were only ~ 14 d apart, with minimal changes in neurodevelopment reported in this period. Our 72 h cohort were also mixed sex, with the females slightly smaller than the males. Adult female ferrets have slightly lower cerebrum volume than males^109^, and males have larger cerebellar cortices from PND10^110^. Nonetheless, clinically, diffusion tractography imaging metrics in slightly older children (8–15) showed no sex related differences in white matter pathology^111^, and pre-clinically no sex related differences were seen in sensorimotor performance including balance on the ledged beam to 28 d post-injury when rats had entered adulthood^112^. In contrast, sex differences in adulthood have been shown following paediatric focal injury in PND21 mice, with males showing deficits in sociability and social recognition, associated with a loss of dendritic complexity in the hippocampus^113^. This may represent domain-specific sex effects that only become apparent when prior brain injury intersects with brain maturation affecting by hormonal changes in adolescence and thus should not have affected motor and cognitive performance to 72 h post-injury here.

This study provides the first comprehensive characterisation of a paediatric ferret model of diffuse TBI, demonstrating extensive axonal injury that could be detected via both APP and NFL and increases in clinically relevant biomarkers, in NFL and GFAP. Axonal injury was associated with subtle motor and cognitive deficits and as such future studies could examine how early-life mild-moderate diffuse TBI interacts with neurodevelopment to affect these domains into adulthood. Ferrets provide an alternative to pre-existing pig models of paediatric TBI, with the advantages of a gyrencephalic brain in a smaller animal to reduce the logistics and cost of study.

## Data Availability

The datasets generated and analysed during the current study, including quantified histological outcomes and behavioural raw data, are available from the corresponding author upon reasonable request, subject to ethical approval and institutional data governance requirements. Due to the size of the imaging files and animal ethics constraints, raw microscopy images and associated metadata will be shared where feasible following appropriate review.

## References

[1] Melichová, J., Majdan, M., Rusnák, M., Zelinková, V. & Taylor, M. Mortality and hospitalization rate due to traumatic brain injury in children, young adults in Europe. *Eur J. Public. Health* **28**. 10.1093/eurpub/cky214.043 (2018).

[2] Schneier, A. J., Shields, B. J., Hostetler, S. G., Xiang, H. & Smith, G. A. Incidence of pediatric traumatic brain injury and associated hospital resource utilization in the united States. *Pediatrics* **118**, 483–492 (2006). 16882799 10.1542/peds.2005-2588

[3] Yeates, K. O. et al. Longitudinal trajectories of postconcussive symptoms in children with mild traumatic brain injuries and their relationship to acute clinical status. *Pediatrics* **123**, 735–743 (2009). 19254996 10.1542/peds.2008-1056 PMC2839361

[4] Taylor, C. A., Bell, J. M., Breiding, M. J. & Xu, L. Traumatic brain Injury-Related emergency department Visits, Hospitalizations, and Deaths - United States, 2007 and 2013. *Morb Mortal. Wkly. Rep. Surveill Summ. Wash. DC 2002*. **66**, 1–16 (2017). 10.15585/mmwr.ss6609a1 PMC5829835 28301451

[5] Bierbaum, M., Lystad, R. P., Curtis, K. & Mitchell, R. Incidence and severity of head injury hospitalisations in Australian children over a 10-year period. *Health Promot J. Austr.* **30**, 189–198 (2019). 30030878 10.1002/hpja.186

[6] Australian Institute of Health and Welfare. *Head Injuries in Australia 2020–21*. (2023). https://www.aihw.gov.au/reports/injury/head-injuries-in-australia-2020-21/data

[7] Ewing-Cobbs, L., Fletcher, J. M., Levin, H. S., Iovino, I. & Miner, M. E. Academic achievement and academic placement following traumatic brain injury in children and adolescents: a two-year longitudinal study. *J. Clin. Exp. Neuropsychol.* **20**, 769–781 (1998). 10484689 10.1076/jcen.20.6.769.1109

[8] Anderson, V., Catroppa, C., Morse, S., Haritou, F. & Rosenfeld, J. Functional plasticity or vulnerability after early brain injury? *Pediatrics* **116**, 1374–1382 (2005). 16322161 10.1542/peds.2004-1728

[9] Hessen, E., Nestvold, K. & Anderson, V. Neuropsychological function 23 years after mild traumatic brain injury: A comparison of outcome after paediatric and adult head injuries. *Brain Inj*. **21**, 963–979 (2007). 17729049 10.1080/02699050701528454

[10] Pinto, P. S., Poretti, A., Meoded, A., Tekes, A. & Huisman, T. A. G. M. The unique features of traumatic brain injury in Children. Review of the characteristics of the pediatric skull and brain, mechanisms of Trauma, patterns of injury, complications and their imaging Findings—Part 1. *J. Neuroimaging*. **22**, e1–e17 (2012). 22303964 10.1111/j.1552-6569.2011.00690.x

[11] Ommaya, A. K., Goldsmith, W. & Thibault, L. Biomechanics and neuropathology of adult and paediatric head injury. *Br. J. Neurosurg.* **16**, 220–242 (2002). 12201393 10.1080/02688690220148824

[12] Sarkar, K. et al. Computed tomography characteristics in pediatric versus adult traumatic brain injury: clinical Article. *J. Neurosurg. Pediatr.* **13**, 307–314 (2014). 24410128 10.3171/2013.12.PEDS13223

[13] Choi, J. I. & Kim, S. D. Pediatric minor traumatic brain injury : growing skull Fracture, traumatic cerebrospinal fluid Leakage, concussion. *J. Korean Neurosurg. Soc.* **65**, 348–353 (2022). 35468709 10.3340/jkns.2021.0280 PMC9082117

[14] Hazwani, T. et al. Diffuse axonal injury on magnetic resonance imaging and its relation to neurological outcomes in pediatric traumatic brain injury. *Clin. Neurol. Neurosurg.* **237**, 108166 (2024). 38364490 10.1016/j.clineuro.2024.108166

[15] Gentry, L., Godersky, J. & Thompson, B. MR imaging of head trauma: review of the distribution and radiopathologic features of traumatic lesions. *Am. J. Roentgenol.* **150**, 663–672 (1988). 3257624 10.2214/ajr.150.3.663

[16] Hellstrøm, T. et al. White matter microstructure is associated with functional, cognitive and emotional symptoms 12 months after mild traumatic brain injury. *Sci. Rep.* **7**, 13795 (2017). 29061970 10.1038/s41598-017-13628-1 PMC5653776

[17] Ware, A. L. et al. Post-acute white matter microstructure predicts post-acute and chronic post-concussive symptom severity following mild traumatic brain injury in children. *NeuroImage Clin.* **25**, 102106 (2020). 31896466 10.1016/j.nicl.2019.102106 PMC6940617

[18] Nwafor, D. C. et al. Pediatric traumatic brain injury: an update on preclinical Models, clinical Biomarkers, and the implications of cerebrovascular dysfunction. *J. Cent. Nerv. Syst. Dis.* **14**, 11795735221098125 (2022). 35620529 10.1177/11795735221098125 PMC9127876

[19] Dietvorst, S., Vervekken, A. & Depreitere, B. Developing a Porcine model of severe traumatic brain injury induced by high amplitude rotational acceleration. *Brain Spine*. **4**, 102728 (2024). 38510621 10.1016/j.bas.2023.102728 PMC10951692

[20] Chen, S., Pickard, J. D. & Harris, N. G. Time course of cellular pathology after controlled cortical impact injury. *Exp. Neurol.* **182**, 87–102 (2003). 12821379 10.1016/s0014-4886(03)00002-5

[21] Adelson, P. D., Robichaud, P., Hamilton, R. L. & Kochanek, P. M. A model of diffuse traumatic brain injury in the immature rat. *J. Neurosurg.* **85**, 877–884 (1996). 8893727 10.3171/jns.1996.85.5.0877

[22] Adelson, P. D., Whalen, M. J., Kochanek, P. M., Robichaud, P. & Carlos, T. M. Blood brain barrier permeability and acute inflammation in two models of traumatic brain injury in the immature rat: a preliminary report. *Acta Neurochir. Suppl.* **71**, 104–106 (1998). 9779157 10.1007/978-3-7091-6475-4_31

[23] Adelson, P. D. et al. Histopathologic response of the immature rat to diffuse traumatic brain injury. *J. Neurotrauma*. **18**, 967–976 (2001). 11686497 10.1089/08977150152693674

[24] Adelson, P. D., Dixon, C. E. & Kochanek, P. M. Long-Term dysfunction following diffuse traumatic brain injury in the immature rat. *J. Neurotrauma*. **17**, 273–282 (2000). 10776912 10.1089/neu.2000.17.273

[25] Huh, J. W., Widing, A. G. & Raghupathi, R. Midline brain injury in the immature rat induces sustained cognitive deficits, bihemispheric axonal injury and neurodegeneration. *Exp. Neurol.* **213**, 84–92 (2008). 18599043 10.1016/j.expneurol.2008.05.009 PMC2633731

[26] DiLeonardi, A. M., Huh, J. W. & Raghupathi, R. Impaired axonal transport and neurofilament compaction occur in seperate populations of injured axons following diffuse brain injury in the immature rat. *Brain Res.* **1263**, 174–182 (2009). 19368848 10.1016/j.brainres.2009.01.021 PMC2696174

[27] Raghupathi, R. & Huh, J. W. Diffuse brain injury in the immature rat: evidence for an Age-at-Injury effect on cognitive function and histopathologic damage. *J. Neurotrauma*. **24**, 1596–1608 (2007). 17970623 10.1089/neu.2007.3790

[28] Dikranian, K. et al. Mild traumatic brain injury to the infant mouse causes robust white matter axonal degeneration which precedes apoptotic death of cortical and thalamic neurons. *Exp. Neurol.* **211**, 551–560 (2008). 18440507 10.1016/j.expneurol.2008.03.012 PMC2486437

[29] Prins, M. L., Lee, S. M., Cheng, C. L. Y., Becker, D. P. & Hovda, D. A. Fluid percussion brain injury in the developing and adult rat: a comparative study of mortality, morphology, intracranial pressure and mean arterial blood pressure. *Dev. Brain Res.* **95**, 272–282 (1996). 8874903 10.1016/0165-3806(96)00098-3

[30] Rowe, R. K. et al. Aging with traumatic brain injury: effects of age at injury on behavioral outcome following diffuse brain injury in rats. *Dev. Neurosci.* **38**, 195–205 (2016). 27449121 10.1159/000446773

[31] Newell, E. A. et al. A mouse model for Juvenile, lateral fluid percussion brain injury reveals Sex-Dependent differences in neuroinflammation and functional recovery. *J. Neurotrauma*. **37**, 635–646 (2020). 31621484 10.1089/neu.2019.6675 PMC7045348

[32] Levchakov, A., Linder-Ganz, E., Raghupathi, R., Margulies, S. S. & Gefen, A. Computational studies of strain exposures in neonate and mature rat brains during closed head impact. *J. Neurotrauma*. **23**, 1570–1580 (2006). 17020491 10.1089/neu.2006.23.1570

[33] Yakovlev, A. G. et al. Differential expression of apoptotic protease-activating factor-1 and caspase-3 genes and susceptibility to apoptosis during brain development and after traumatic brain injury. *J. Neurosci. Off J. Soc. Neurosci.* **21**, 7439–7446 (2001). 10.1523/JNEUROSCI.21-19-07439.2001 PMC6762901 11567033

[34] Bodnar, C. N., Roberts, K. N., Higgins, E. K. & Bachstetter, A. D. A systematic review of closed head injury models of mild traumatic brain injury in mice and rats. *J. Neurotrauma*. **36**, 1683–1706 (2019). 30661454 10.1089/neu.2018.6127 PMC6555186

[35] Sorby-Adams, A. J., Vink, R. & Turner, R. J. Large animal models of stroke and traumatic brain injury as translational tools. *Am. J. Physiol. -Regul Integr. Comp. Physiol.* **315**, R165–R190 (2018). 29537289 10.1152/ajpregu.00163.2017

[36] Cloots, R. J., Gervaise, H. M., van Dommelen, J. A. & Geers, M. G. Biomechanics of traumatic brain injury: influences of the morphologic heterogeneities of the cerebral cortex. *Ann. Biomed. Eng.* **36**, 1203–1215 (2008). 18465248 10.1007/s10439-008-9510-3 PMC2413127

[37] Ibrahim, N. G., Ralston, J., Smith, C. & Margulies, S. S. Physiological and pathological responses to head rotations in toddler piglets. *J. Neurotrauma*. **27**, 1021–1035 (2010). 20560753 10.1089/neu.2009.1212 PMC2943503

[38] Sullivan, S. et al. Improved Behavior, Motor, and cognition assessments in neonatal piglets. *J. Neurotrauma*. **30**, 1770–1779 (2013). 23758416 10.1089/neu.2013.2913 PMC3796335

[39] Weeks, D., Sullivan, S., Kilbaugh, T., Smith, C. & Margulies, S. S. Influences of developmental age on the resolution of diffuse traumatic intracranial hemorrhage and axonal injury. *J. Neurotrauma*. **31**, 206–214 (2014). 23984914 10.1089/neu.2013.3113 PMC3901955

[40] Kilbaugh, T. J. et al. Mitochondrial response in a toddler-aged swine model following diffuse non-impact traumatic brain injury. *Mitochondrion* **26**, 19–25 (2016). 26549476 10.1016/j.mito.2015.11.001 PMC4752861

[41] Raghupathi, R. & Margulies, S. S. Traumatic axonal injury after closed head injury in the neonatal pig. *J. Neurotrauma*. **19**, 843–853 (2002). 12184854 10.1089/08977150260190438

[42] Friess, S. H. et al. Neurobehavioral functional deficits following closed head injury in the neonatal pig. *Exp. Neurol.* **204**, 234–243 (2007). 17174304 10.1016/j.expneurol.2006.10.010 PMC1892165

[43] Eucker, S., Smith, C., Ralston, J., Friess, S. & Margulies, S. Physiological and histopathological responses following closed rotational head injury depend on direction of head motion. *Exp. Neurol.* **227**, 79–88 (2011). 20875409 10.1016/j.expneurol.2010.09.015 PMC3021173

[44] Ibrahim, N. G., Wood, J., Margulies, S. S. & Christian, C. W. Influence of age and fall type on head injuries in infants and toddlers. *Int. J. Dev. Neurosci.* **30**, 201–206 (2012). 22079853 10.1016/j.ijdevneu.2011.10.007 PMC3288448

[45] Huber, C. M., Thakore, A. D., Oeur, R. A. & Margulies, S. S. Distinct Serum Glial Fibrillary Acidic Protein and Neurofilament Light Time-Courses After Rapid Head Rotations. *J. Neurotrauma* neu.2023.0660. 10.1089/neu.2023.0660 (2024). 10.1089/neu.2023.0660 PMC11564843 38698671

[46] Jaber, S. M., Sullivan, S. & Margulies, S. S. Noninvasive metrics for identification of brain injury deficits in piglets. *Dev. Neuropsychol.* **40**, 34–39 (2015). 25649778 10.1080/87565641.2014.969733 PMC4318352

[47] Vink, R. Large animal models of traumatic brain injury. *J. Neurosci. Res.* **96**, 527–535 (2018). 28500771 10.1002/jnr.24079

[48] Gilardi, C. & Kalebic, N. The ferret as a model system for neocortex development and evolution. *Front Cell. Dev. Biol* **9**. 10.3389/fcell.2021.661759 (2021). 10.3389/fcell.2021.661759 PMC8118648 33996819

[49] Wood, T. et al. A ferret model of encephalopathy of prematurity. *Dev. Neurosci.* **40**, 475–489 (2018). 31079096 10.1159/000498968 PMC6658350

[50] Danka Mohammed, C. P. & Khalil, R. Postnatal development of visual cortical function in the mammalian brain. *Front Syst. Neurosci* **14**. 10.3389/fcell.2021.661759 (2020). 10.3389/fnsys.2020.00029 PMC7296053 32581733

[51] Semple, B. D., Blomgren, K., Gimlin, K., Ferriero, D. M. & Noble-Haeusslein, L. J. Brain development in rodents and humans: identifying benchmarks of maturation and vulnerability to injury across species. *Prog Neurobiol.* **0**, 1–16 (2013). 10.1016/j.pneurobio.2013.04.001 PMC3737272 23583307

[52] Krieg, J. L., Leonard, A. V., Tuner, R. J. & Corrigan, F. Characterization of traumatic brain injury in a gyrencephalic ferret model using the novel closed head injury model of engineered rotational acceleration (CHIMERA). *Neurotrauma Rep.* **4**, 761–780 (2023). 38028274 10.1089/neur.2023.0047 PMC10659026

[53] Krieg, J. L. et al. Evolution of axonal injury in the closed head impact model of engineered rotational acceleration in adult ferrets. *J. Neurosci. Res.* **103**, e70090 (2025). 41163442 10.1002/jnr.70090

[54] Seibenhener, M. L. & Wooten, M. C. Use of the open field maze to measure locomotor and Anxiety-like behavior in mice. *J. Vis. Exp. JoVE*. **52434**10.3791/52434 (2015). 10.3791/52434 PMC4354627 25742564

[55] Ben Abdallah, N. M. B. et al. The puzzle box as a simple and efficient behavioral test for exploring impairments of general cognition and executive functions in mouse models of schizophrenia. *Exp. Neurol.* **227**, 42–52 (2011). 20851119 10.1016/j.expneurol.2010.09.008

[56] Osterman, C. et al. Perivascular glial reactivity is a feature of phosphorylated Tau lesions in chronic traumatic encephalopathy. *Acta Neuropathol. (Berl)*. **149**, 16 (2025). 39921702 10.1007/s00401-025-02854-x PMC11807024

[57] Bankhead, P. et al. QuPath: open source software for digital pathology image analysis. *Sci. Rep.* **7**, 16878 (2017). 29203879 10.1038/s41598-017-17204-5 PMC5715110

[58] O’Brien, W. T. et al. Temporal profile and utility of serum neurofilament light in a rat model of mild traumatic brain injury. *Exp. Neurol.* **341**, 113698 (2021). 33727100 10.1016/j.expneurol.2021.113698

[59] Brooks, T. et al. Finite element models and material data for analysis of infant head impacts. *Heliyon* **4**, e01010 (2018). 30582038 10.1016/j.heliyon.2018.e01010 PMC6288411

[60] Powell, E. C. et al. Isolated linear skull fractures in children with blunt head trauma. *Pediatrics* **135**, e851–e857 (2015). 25780067 10.1542/peds.2014-2858

[61] Erlichman, D. B., Blumfield, E., Rajpathak, S. & Weiss, A. Association between linear skull fractures and intracranial hemorrhage in children with minor head trauma. *Pediatr. Radiol.* **40**, 1375–1379 (2010). 20217069 10.1007/s00247-010-1555-4

[62] Donaldson, K., Li, X., Sartorelli, K. H., Weimersheimer, P. & Durham, S. R. Management of isolated skull fractures in pediatric patients: A systematic review. *Pediatr. Emerg. Care*. **35**, 301–308 (2019). 30855424 10.1097/PEC.0000000000001814

[63] Khanna, S. K., Kumar, A., Katiyar, A. K. & Mishra, K. Clinical profile, management, and outcome of pediatric neurotrauma: a multicentric observational study. *J. Trauma. Inj*. **38**, 22–31 (2025). 40175077 10.20408/jti.2024.0080 PMC11968312

[64] Sim, S. Y. et al. Reappraisal of pediatric diastatic skull fractures in the 3-Dimensional CT era: clinical characteristics and comparison of diagnostic accuracy of simple skull X-Ray, 2-Dimensional CT, and 3-Dimensional CT. *World Neurosurg.* **108**, 399–406 (2017). 28844920 10.1016/j.wneu.2017.08.107

[65] Shan, J. et al. Computed tomography use in children with minor head trauma presenting to 21 community emergency departments within an integrated Health-Care system. *Perm J.* **26**, 32–37 (2022). 10.7812/TPP/21.096 PMC9126554 35609173

[66] Marin, J. R. et al. Variation in emergency department head computed tomography use for pediatric head trauma. *Acad. Emerg. Med. Off J. Soc. Acad. Emerg. Med.* **21**, 987–995 (2014). 10.1111/acem.12458 25269579

[67] Anbalagan, P., Jamal, B. C., Saqib, H. & Ganti, L. Predictors of skull fracture and intracerebral pathology after pediatric traumatic brain injury. *Orthop Rev* **17**. 10.52965/001c.137676 (2025). 10.52965/001c.137676 PMC12085273 40384922

[68] Gefen, A., Gefen, N., Zhu, Q., Raghupathi, R. & Margulies, S. S. Age-dependent changes in material properties of the brain and braincase of the rat. *J. Neurotrauma*. **20**, 1163–1177 (2003). 14651804 10.1089/089771503770802853

[69] Krieg, J. L., Leonard, A. V., Turner, R. J. & Corrigan, F. Identifying the phenotypes of diffuse axonal injury following traumatic brain injury. *Brain Sci.* **13**, 1607 (2023). 38002566 10.3390/brainsci13111607 PMC10670443

[70] Leermakers, F. M. & Zhulina, E. B. How the projection domains of NF-L and alpha-internexin determine the conformations of NF-M and NF-H in neurofilaments. *Eur. Biophys. J. EBJ*. **39**, 1323–1334 (2010). 20213320 10.1007/s00249-010-0585-z

[71] Dileonardi, A. M., Huh, J. W. & Raghupathi, R. Differential effects of FK506 on structural and functional axonal deficits after diffuse brain injury in the immature rat. *J. Neuropathol. Exp. Neurol.* **71**, 959–972 (2012). 23095847 10.1097/NEN.0b013e31826f5876 PMC3495060

[72] Zhou, Z., Li, X. & Kleiven, S. Biomechanics of periventricular injury. *J. Neurotrauma*. **37**, 1074–1090 (2020). 31701809 10.1089/neu.2019.6634 PMC7185329

[73] Tang-Schomer, M. D., Patel, A. R., Baas, P. W. & Smith, D. H. Mechanical breaking of microtubules in axons during dynamic stretch injury underlies delayed elasticity, microtubule disassembly, and axon degeneration. *FASEB J. Off Publ Fed. Am. Soc. Exp. Biol.* **24**, 1401–1410 (2010). 10.1096/fj.09-142844 PMC2879950 20019243

[74] Viancour, T. A. & Kreiter, N. A. Vesicular fast axonal transport rates in young and old rat axons. *Brain Res.* **628**, 209–217 (1993). 8313149 10.1016/0006-8993(93)90957-o

[75] Song, H. et al. Traumatic brain injury recapitulates developmental changes of axons. *Prog Neurobiol.* **217**, 102332 (2022). 35870679 10.1016/j.pneurobio.2022.102332 PMC9454890

[76] Weber, J. T. Altered calcium signaling following traumatic brain injury. *Front. Pharmacol.* **3**, 60 (2012). 22518104 10.3389/fphar.2012.00060 PMC3324969

[77] Shaw, G. et al. Uman-type neurofilament light antibodies are effective reagents for the imaging of neurodegeneration. *Brain Commun.* **5**, fcad067 (2023). 37091583 10.1093/braincomms/fcad067 PMC10120172

[78] Fusco, A. F. et al. Immunohistochemical labeling of ongoing axonal degeneration 10 days following cervical contusion spinal cord injury in the rat. *Spinal Cord*. **63**, 86–94 (2025). 39753895 10.1038/s41393-024-01053-x PMC11849397

[79] Fusco, A. F. et al. Serum evaluation of NFL correlates with histological identification of degenerating axons. *Exp. Neurol.* **392**, 115360 (2025). 40562346 10.1016/j.expneurol.2025.115360 PMC12782307

[80] Gallyas, F., Pál, J., Farkas, O. & Dóczi, T. The fate of axons subjected to traumatic ultrastructural (neurofilament) compaction: an electron-microscopic study. *Acta Neuropathol. (Berl)*. **111**, 229–237 (2006). 16485106 10.1007/s00401-006-0034-3

[81] Pant, H. C. Dephosphorylation of neurofilament proteins enhances their susceptibility to degradation by Calpain. *Biochem. J.* **256**, 665–668 (1988). 2851997 10.1042/bj2560665 PMC1135461

[82] Fournier, A. et al. Changes in neurofilament and microtubule distribution following focal axon compression. *PLoS One*. **10**, e0131617 (2015). 26111004 10.1371/journal.pone.0131617 PMC4482325

[83] Munoz Pareja, J. C. et al. Prognostic and diagnostic utility of serum biomarkers in pediatric traumatic brain injury. *J. Neurotrauma*. **41**, 106–122 (2024). 37646421 10.1089/neu.2023.0039 PMC11071081

[84] Hellewell, S., Yan, E., Agyapomaa, D., Bye, N. & Morganti-Kossmann, M. Post-traumatic hypoxia exacerbates brain tissue damage: analysis of axonal injury and glial responses. *J. Neurotrauma*. **27**, 1997–2010 (2010). 20822466 10.1089/neu.2009.1245

[85] Kelley, B. J., Lifshitz, J. & Povlishock, J. T. Neuroinflammatory responses after experimental diffuse traumatic brain injury. *J. Neuropathol. Exp. Neurol.* **66**, 989–1001 (2007). 17984681 10.1097/NEN.0b013e3181588245

[86] Oehmichen, M., Theuerkauf, I. & Meissner, C. Is traumatic axonal injury (AI) associated with an early microglial activation? Application of a double-labeling technique for simultaneous detection of microglia and AI. *Acta Neuropathol. (Berl)*. **97**, 491–494 (1999). 10334486 10.1007/s004010051018

[87] Smith, C. et al. The neuroinflammatory response in humans after traumatic brain injury. *Neuropathol. Appl. Neurobiol.* **39**10.1111/nan.12008 (2013). 10.1111/nan.12008 PMC3833642 23231074

[88] Green, T. R. F., Murphy, S. M., Ortiz, J. B. & Rowe, R. K. Age-At-Injury influences the glial response to traumatic brain injury in the cortex of male juvenile rats. *Front. Neurol.* **12**, 804139 (2022). 35111130 10.3389/fneur.2021.804139 PMC8802670

[89] Green, T. R. F. et al. Blood–Brain barrier dysfunction predicts microglial activation after traumatic brain injury in juvenile rats. *Neurotrauma Rep.* **5**, 95–116 (2024). 38404523 10.1089/neur.2023.0057 PMC10890961

[90] Schafer, D. P. et al. Microglia sculpt postnatal neural circuits in an activity and complement-dependent manner. *Neuron* **74**, 691–705 (2012). 22632727 10.1016/j.neuron.2012.03.026 PMC3528177

[91] Hanlon, L. A., Raghupathi, R. & Huh, J. W. Depletion of microglia immediately following traumatic brain injury in the pediatric rat: implications for cellular and behavioral pathology. *Exp. Neurol.* **316**, 39–51 (2019). 30980832 10.1016/j.expneurol.2019.04.004 PMC6544393

[92] Fraser, D. D. et al. Severe traumatic brain injury in children elevates glial fibrillary acidic protein in cerebrospinal fluid and serum. *Pediatr. Crit. Care Med. J. Soc. Crit. Care Med. World Fed. Pediatr. Intensive Crit. Care Soc.* **12**, 319–324 (2011). 10.1097/PCC.0b013e3181e8b32d 20625342

[93] Ryan, E. et al. Traumatic brain injury in children: glial fibrillary acidic protein and clinical outcomes. *Pediatr. Emerg. Care*. **38**, e1139–e1142 (2022). 34469402 10.1097/PEC.0000000000002527

[94] Žurek, J. & Fedora, M. Dynamics of glial fibrillary acidic protein during traumatic brain injury in children. *J. Trauma. Acute Care Surg.* **71**, 854 (2011). 10.1097/TA.0b013e3182140c8c 21986734

[95] Kayhanian, S. et al. Thresholds for identifying pathological intracranial pressure in paediatric traumatic brain injury. *Sci. Rep.* **9**, 3537 (2019). 30837528 10.1038/s41598-019-39848-1 PMC6401127

[96] Esparza, J. et al. Outcome in children with severe head injuries. *Childs Nerv. Syst. ChNS Off J. Int. Soc. Pediatr. Neurosurg.* **1**, 109–114 (1985). 10.1007/BF00706691 4005881

[97] Dupont, D. et al. Postconcussive symptoms after early childhood concussion. *JAMA Netw. Open.* **7**, e243182 (2024). 38512252 10.1001/jamanetworkopen.2024.3182 PMC10958232

[98] Caeyenberghs, K. et al. Correlations between white matter integrity and motor function in traumatic brain injury patients. *Neurorehabil Neural Repair.* **25**, 492–502 (2011). 21427274 10.1177/1545968310394870

[99] Caeyenberghs, K. et al. Brain-behavior relationships in young traumatic brain injury patients: DTI metrics are highly correlated with postural control. *Hum. Brain Mapp.* **31**, 992–1002 (2010). 19998364 10.1002/hbm.20911 PMC6870903

[100] Peters, B. D. et al. Age-Related differences in white matter tract microstructure are associated with cognitive performance from childhood to adulthood. *Biol. Psychiatry*. **75**, 248–256 (2014). 23830668 10.1016/j.biopsych.2013.05.020 PMC4412928

[101] Nagy, Z., Westerberg, H. & Klingberg, T. Maturation of white matter is associated with the development of cognitive functions during childhood. *J. Cogn. Neurosci.* **16**, 1227–1233 (2004). 15453975 10.1162/0898929041920441

[102] Dremmen, M. H. G. et al. Cavum septum pellucidum in the general pediatric population and its relation to surrounding brain structure Volumes, cognitive Function, and emotional or behavioral problems. *Am. J. Neuroradiol.* **40**, 340–346 (2019). 30679220 10.3174/ajnr.A5939 PMC7028615

[103] Douet, V. & Chang, L. Fornix as an imaging marker for episodic memory deficits in healthy aging and in various neurological disorders. *Front Aging Neurosci* **6**. 10.3389/fnagi.2014.00343 (2015). 10.3389/fnagi.2014.00343 PMC4294158 25642186

[104] Mayer, A. R. et al. Diffusion abnormalities in pediatric mild traumatic brain injury. *J. Neurosci.* **32**, 17961–17969 (2012). 23238712 10.1523/JNEUROSCI.3379-12.2012 PMC6621719

[105] Wu, Y. et al. Mild traumatic brain injury induces microvascular injury and accelerates Alzheimer-like pathogenesis in mice. *Acta Neuropathol. Commun.* **9**, 74 (2021). 33892818 10.1186/s40478-021-01178-7 PMC8063402

[106] Dennis, E. L. et al. Callosal function in pediatric traumatic brain injury linked to disrupted white matter integrity. *J. Neurosci.* **35**, 10202–10211 (2015). 26180196 10.1523/JNEUROSCI.1595-15.2015 PMC4502260

[107] D’Mello, V. et al. Intranasal leukemia inhibitory factor attenuates gliosis and axonal injury and improves sensorimotor function after a mild pediatric traumatic brain injury. *Neurotrauma Rep.* **4**, 236–250 (2023). 37095853 10.1089/neur.2021.0075 PMC10122240

[108] Kinder, H. A., Baker, E. W., Howerth, E. W., Duberstein, K. J. & West, F. D. Controlled cortical impact leads to cognitive and motor function deficits that correspond to cellular pathology in a piglet traumatic brain injury model. *J. Neurotrauma*. **36**, 2810–2826 (2019). 31084390 10.1089/neu.2019.6405

[109] Sawada, K., Horiuchi-Hirose, M., Saito, S. & Aoki, I. MRI-based morphometric characterizations of sexual dimorphism of the cerebrum of ferrets (Mustela putorius). *NeuroImage* **83**, 294–306 (2013). 23770407 10.1016/j.neuroimage.2013.06.024

[110] Sawada, K. & Aoki, I. Age-Dependent Sexually-Dimorphic asymmetric development of the ferret cerebellar cortex. *Symmetry* **9**, 40 (2017).

[111] Goodrich-Hunsaker, N. J. et al. Age and sex-related effects in children with mild traumatic brain injury on diffusion MRI properties: a comparison of Voxelwise and tractography methods. *J. Neurosci. Res.* **96**, 626–641 (2018). 28984377 10.1002/jnr.24142 PMC5803411

[112] Russell, K. L., Kutchko, K. M., Fowler, S. C., Berman, N. E. J. & Levant, B. Sensorimotor behavioral tests for use in a juvenile rat model of traumatic brain injury: assessment of sex differences. *J. Neurosci. Methods*. **199**, 214–222 (2011). 21600923 10.1016/j.jneumeth.2011.05.008 PMC3142868

[113] Semple, B. D., Canchola, S. A. & Noble-Haeusslein, L. J. Deficits in social behavior emerge during development after pediatric traumatic brain injury in mice. *J. Neurotrauma*. **29**, 2672–2683 (2012). 22888909 10.1089/neu.2012.2595 PMC3510450

